# Analectic

**Published:** 1835

**Authors:** 


### MISCELLANEOUS INTELLIGENCE.

### ANALECTIC, ANALYTICAL, AND ORIGINAL.

### I. Analectic.

*Ad Sylvas Nuncius.*

1. *Theorectical and Practical Treatise on Wounds inflicted by the Arms of War.** By M. Dupuytren.—Few surgeons have been so successful in their professional career as M. Dupuytren, and none have acquired a more brilliant reputation. Possessed of excellent judgment, a retentive memory, and great zeal and enthusiasm in his professional pursuits, he added to these qualities, a remarkable penetration, a quickness of perception seldom witnessed, and which admirably fitted him for the investigation of diseases. And for this purpose, no one perhaps ever enjoyed greater opportunities. Entrusted with the charge of one of the most extensive hospitals in Europe; enjoying moreover a large share of private practice,—he could not fail to be familiar with the nature, and various forms of disease;—and it was particularly at the bedside of the patient, that his superiority in diagnosis, and excellence as a practitioner, were strikingly manifest.

Among the different classes of disease or injury, which the surgeon is called upon to treat, there is one, the entire knowledge of which cannot be obtained in time of peace, neither in the quiet walks of the hospital, nor in the gradual course of private practice, but must be sought among the horrors of war, and scenes of death and bloodshed. It seldom falls to the lot of surgeons at home, to have opportunities of witnessing the effects produced by arms of war. The war, however, of 1814–'15, and the celebrated revolution of July, 1830, afforded to M. Dupuytren these advantages;—the wounded were received into the hotel dieu, and placed under his guidance and direction. It was from the observation of these numerous cases of injury, that the principles contained in the work before us were derived; and to those, in fact, it owes it origin. It is indeed a series of lectures delivered by M. Dupuytren, at the request of several

*Traite Theorique et Pratique des blessures par Armes de Guerre, redige d'apres les lecons cliniques de M. Le Baron Dupuytren, Chirurgien en chef de l'Hotel Dieu, et publie sous sa direction par M. M. less Docteurs A. Paillard et Marx, 2 tome, pp. 522 and 527, Paris, 1834.

As this work has not reached us we avail ourselves of a notice of its contents as given by one of our respected cotemporaries.—Ed. West, Jour.

physicians and surgeons, as well as students and strangers. The work itself consists of two volumes, the first of which is taken up with the consideration of wounds in general, without reference to the particular part or region in which they may be inflicted. It is divided into twelve chapters. The 1st is devoted to a description of the different weapons or arms of war, which are in modern use, and contains much that is curious and interesting, particularly to the military surgeon. We shall omit this, however, and pass to the consideration of the effects produced by these agents—subjects with which we are more particularly concerned.

The effects produced by arms of war are exceedingly various, and must depend, of course, upon the nature of the weapon, the force with which the injury may be inflicted, and the nature of the organ or part concerned.

As almost every thing has been applied to the purposes of war, and may be used as a weapon, offensive or defensive, the appearance and the nature of wounds, produced by these agents vary exceedingly; some inflicting injury, by division of the tissues or by hemorrhage; others by violently bruising or tearing the parts, etc. Hence, M. Dupuytren has made a division of wounds into—"1st, punctured—2d, incised—3d, wounds by puncture and division—4th, contused wounds—5th, wounds by commotion—6th, wounds by crushing of parts, (*ecrasement*)—7th, lacerated wounds—8th, wounds par arrachement—9th, wounds by attrition, i. e. such as are caused by bodies propelled by the force of air or steam—10th, wounds produced by small fire-arms, as guns, pistols, etc.—11th, wounds produced by cannon balls, etc.—12th, wounds produced by gunpowder.

In wounds produced *par arrachement*, there is laceration produced simply by excessive extension: or this may be united with torsion. The cases of limbs torn off by machinery will illustrate this. We have used the word in this place, as its meaning could not be fully expressed by another in English. Under the head of punctured wounds, M. Dupuytren alludes to the effect produced by round and pointed instruments, as bodkins, spindles, etc. The form of the wound inflicted by these instruments seems not to have attracted attention, although, from its singularity and difference of character from that which has generally been supposed, it is worthy the attention of the medical jurist. From several observations made upon the living and the dead subject, M. Dupuytren has ascertained, that wounds made by these instruments are similar, in almost every respect, to those inflicted by cutting weapons. The borders of the wound are even and nearly in apposition, and if they are separated, slight tension of the skin will effect their union;—punctures of this nature always have the same direction in the same region. In the neck, and on the anterior part of the axilla, the direction of the puncture is from above, downward. On the thorax, they are parallel to the direction of the ribs, or the intercostal spaces. On the anterior part of the abdomen, they are oblique from above downwards, and seem to assume the direction of the muscular fibre. In the middle of this region they are vertical, while on the members they are parallel to the axis of the limb. These facts appear to show, clearly, the different direction of the fibres of the skin in the different regions, and the instrument seems to act merely by separating them.

Case.—"Charles Levauvre, aged, 33, strong constitution and sanguine temperament, attempted suicide by stabbing himself three times in the region of the heart with a pointed bodkin. When taken to the hotel dieu, three small wounds of two lines in length were observed opposite the 7th rib. The lips of the wound were equal and very near each other, and the angles of it very acute. They were parallel with the rib, and resembled those made with a penknife. The weapon was procured and several punctures made on the dead subject, which presented the same appearance.

In wounds of the hollow organs, the injury produced, varies greatly, according to the state of emptiness or distension, and our conduct must vary accordingly. Should the weapon which has inflicted the injury be still retained in the wound, (suppose it were the bladder,) the urine should first be drawn off by the catheter, before the weapon is removed, otherwise the sudden withdrawing of the instrument might suffer the urine to escape into the cavity of the abdomen, and produce the most intense inflammation and even death. Should it be in the heart, it would be necessary, in the first place, to diminish the quantity of circulating fluid by v.s. Many cases are on record, of wounds of this organ, in which the patient recovered. Its structure, indeed, is well adapted to secure it against the usual consequence of puncture of the hollow organ,—the loss of blood being prevented by the oblique arrangement of its fibres, as well as their contraction, which is sure to ensue.

In speaking of wounds of the articulations, after enjoining the usual rigorous antiphlogistic treatment, M. Dupuytren recommends the application of a blister around and upon the wound itself; and speaks of this treatment as peculiar to Fleury De Clermont. It may be new in France, but the practice is familiar enough in this country, and was we believe, first recommended by Dr. Physic.

Foreign bodies although, for the most part exciting inflammation in the organs in which they are lodged, do, it is well known, often remain in the system for years, without producing any or very serious inconvenience. This is the case with some even, which are very sharp and pointed; and of this M. Dupuytren relates a curious instance: "An officer, with a view to commit suicide, inserted in the region of the heart, one of the long black pins, known by name of *epingle a friser*. This pin penetrated the pericardium and heart, and remained in this viscus during life time. After death the pin was found, the officer having died of another disease."

M. Dupuytren has often had occasion to observe the condition in which foreign bodies exist in the different parts of the body. "Whenever they excite inflammation and suppuration, they are enveloped by a purulent cyst of the nature of mucous membrane, and, at the end of a certain time, a fistulous communication is formed between them and some point of the surface or interior of the body. On the contrary, when they excite neither inflammation nor suppuration, we find them surrounded by a cyst, the form of which corresponds to their own, but whose organization is similar to those of the serous membranes. In their cavity is constantly observed a limpid serosity, analogous to that which lubricates the serous membranes. This remark is not only curious, but important, with regard to practice. If, indeed, in extracting these foreign bodies, we remove them only after making a simple incision of the cyst, and then close the wound, in order to obtain union by the first intention, we find almost always a new tumor produced, by the quantity of serum developed in the sac. It is necessary then, when extracting balls to remove the cyst which surrounds them, or to fill it with charpie, in order to excite inflammation, and obtain adhesion.

But although foreign bodies sometimes remain at rest in the tissues, and give rise to little or no irritation, it is well known that they often travel an immense space of the surface, and even penetrate deeply the interior of the viscera. Particularly does this apply to sharp and pointed substances, as pins, needles, etc. If they traverse the body rapidly, they leave no trace of their passage through the organs. On the contrary, if their passage is slow, they are surrounded by an adventitious serous membrane. In general, they have a tendency from the interior to the external surface. Sometimes, however, this is reversed; but even then there is a strong determination to the interior of the intestinal canal, which in fact may be considered as a continuation of the external surface. It is an interesting fact, remarked by M. Dupuytren, that substances traversing the serous membranes never fall into the interior of these sacs, whereas they frequently, after piercing the serous tissue, penetrate into the mucous cavities. Thus pins which have been swallowed, have been known to pierce the bladder, and being there retained, give rise to the symptoms of stone, and actual formation of a calculus. Of this M. Dupuytren cites an instance.] The above cases show a most beautiful provision of nature. We see these substances almost by instinct, as it were, shunning the serous cavities, where they could not fail to excite inflammation, and all its pernicious consequences, and tending towards the mucous surfaces, which either readily allow their elimination, or at least support their presence with less irritation. There is a singular and almost incredible propensity among some females, to swallow pins, needles, and other sharp and pointed bodies. M. Dupuytren has remarked several cases of this kind in the hotel dieu, and among others, that of a female, who swallowed an enormous quantity of pins and needles, and was reduced by the suffering and pain occasioned by their progress through the skin to a great degree of emaciation. He has opened more than a hundred abscesses, on the body of this female, in which there were always one or two needles or pins. Hardly any movement could be made, without experiencing the greatest suffering. The patient finally died, and on opening the body, many hundreds of pins and needles were found scattered through all the different parts of the body.

We shall pass over the chapters devoted to the consideration of incised wounds, as they do not present any thing of peculiar interest, with the exception, perhaps, of a few pages on wounds, with complete separation of parts. These injuries, when produced by instruments which tear, bruise, or disorganize the structures to any considerable extent, are incapable of reunion; neither can this be expected, when the member is of considerable size. Here the part is too depending on the general system, to retain its vitality sufficiently long, and even were it united to it, as it could only be restored by the action of the capillaries, the extent of this vital action would be too limited, and the part which was separated must die before sufficient circulation and innervation could be established. It is, therefore, only in parts which are small and have been recently separated from the body, that union can be effected. The more speedily this is attempted after the injury, the greater chance is there of success; but it is probable that it may be accomplised at any time, so long as the heat of the part remains, and the blood continues fluid, thus manifesting the existence of the vital properties. Thus M. Dupuytren relates the case of a physician who, in extracting a tooth from a child, by mistake drew out one of the permanent teeth. Discovering this, he endeavored to find the tooth. It had fallen on the floor, and more than an hour elapsed before it was found. By the advice of M. Dupuytren, it was again replaced; the tooth lived, and, at the end of eighteen months, was as firm and useful as the others. Similar facts have not unfrequently been observed. In this case, the vitality of the part remained, probably, much longer than usual. It is well known that parts, which are naturally at great distances from each other, may sometimes be united, as the *Talicotian* operation fully exemplifies. Accidental defects of one individual may sometimes be supplied by another. There have not been wanting individuals kind enough to supply their neighbors with integuments sufficient to repair their noses, when destroyed by accident or disease. The experiments of Hunter are familiar to most readers, and we have seen the spur of a cock which had been transplanted to his head, and had attained the length of two or three inches. M. Paillard and Marx, alluded to some very interesting experiments of this kind performed by M. Baronia.

*Wounds made by Incision and Puncture.*—The character of these wounds may easily be inferred from that of the instruments by which they are inflicted, which having a sharp point, are well calculated for plunging profoundly into the different parts of the body, while by their cutting edge they divide the tissues to a considerable extent. From this it results that there is more hemorrhage; the nerves are more frequently divided, and from the division of the muscles, hernia is more apt to ensue, than in cases of punctured wounds. Sometimes, however, it happens, that the viscera, particularly those of the abdomen, some of which are smooth, loose, and yielding, evade the weapon which pierces this cavity. M. Dupuytren relates an interesting case: "M. N. under the influence of domestic affliction, resolved to destroy himself. To accomplish this, he placed the handle of his sword against the wall of his chamber, and precipitated himself against its point. It passed entirely through the abdomen;—M. Dupuytren was called, and found him seated on a chair; the point of the sword eight or ten inches from the back, and upon the right side of the vertebral column. The patient appeared to suffer very little; there was no sign of effusion of blood or any other fluid into the abdomen cavity, nor of any lesion of the viscera. M. Dupuytren extracted the weapon, not, however, without some difficulty, and a fear that hemorrhage, which had been prevented by the presence of the sword, might occur as soon as it was withdrawn. There was none however; and the patient, after three or four bleedings and a few days of repose, was entirely cured."

Upon this part we shall not dwell, but pass to the consideration of a state of the system which is often found to accompany gun shot wounds; and as its consideration is of great importance, and has been somewhat neglected by authors, we shall speak of it somewhat in detail. This affection is generally the consequence of a severe attrition or contusion of some part, and is called stupor, (*stupeur.*) When this state exists, the sensibility at the seat of injury is greatly diminished; so much so, that it can frequently be amputated without the consciousness of the patient, who is indifferent to all that is taking place about him. In these cases, the heat of the body is greatly reduced, and the limbs are sometimes of icy coldness. A deadly shock has been inflicted on the system, and all the organs are struck with torpor. The heart acts feebly and irregularly, the breathing is slow, the eyes are fixed, the mouth open, and the countenance has an air of astonishment. While this state exists, the wounds do not bleed, but, on the contrary, present a pale or violet color, and are soft and flabby. After a few days, the parts on which the patient reposes, as the great trochanter, sacrum, etc., often ulcerate. If re-action comes on, the wounds become inflamed, and furnish an exhalation from their surface, violet, sanguinolent, or fetid, and the symptoms of stupor disappear, an emphysematous swelling takes place, and extends to the trunk. The fever which ensues is extremely irregular, and is composed of alternate chills and flushes of heat: fierce delirium, vomiting, and jaundice often ensue, together with suppression of urine. Generally, when it has arrived at this state, the patient soon expires. It would appear that, in these cases, there is such an exhaustion and disturbance of nervous energy, that the parts are rendered permanently incapable of resuming their office; hence, the irregularity of the phenomena which ensue. That the vitality of the parts is in a measure destroyed, would seem, moreover, to be manifest from the changes which ensue after death. Thus the body, and in particular that part which has been injured, has been observed to putrify with great rapidity, and decomposition, was far advanced when it had only commenced in the bodies of those who had died of ordinary diseases.

In every case of stupor, where the schock has not too profoundly prostrated the powers of life, we can distinguish two stages: 1st, one of depression which gradually passes into the second, or that of re-action. In the first, we should endeavor to support the powers of life as much as possible by the administration of stimulants and cordials, varying them, however, according to the particular circumstances of the case. In some instances, where the stupor is slight, we should be particularly cautious in giving stimulants, as in many, when the re-action is developed, have cause to regret their too liberal administration. The wound should be first fomented with wine, spirits, or aromatic liquors. In fact, all prudent endeavors should be made to excite the system, and relieve it from the great exhaustion and debility under which it labors; and when re-action has taken place, it is necessary carefully to watch the symptoms as they rise; and our treatment must vary with them. Should much inflammation supervene, it must be subdued by the usual antiphlogistic means; but our bleedings should rather be small, and frequently repeated. If nervous symptoms ensue, antispasmodics are indicated. It is necessary, of course, to remove the patient as much as possible from the pernicious influence of moral causes. M. Dupuytren adds, that those who have suffered from stupor, are more exposed than others to the dangers of the ataxic affections.

*Wounds by Air and Steam Guns.*—These are seldom employed in war—as they possess many inconveniences, and their force of projection is not so great as that of gundowder. The air gun, however, is sometimes used as an instrument of murder, where it is desired to avoid detection. This is rendered difficult, by the absence of light and noise on the occurrence of the discharge; and for this reason they have been prohibited in certain countries. Where a wound has been maliciously and secretly inflicted by one of these instruments, it may be desirable to know, if any criteria exist by which it may be distinguished from one produced by fire arms. The wounds of the latter are often soiled and blackened by the product of the decomposition of the powder, especially the charcoal. This, moreover, is often perceived on the clothes of the wounded, and it may be accompanied by the presence of the wadding. The form and nature of the wound is the same, but there is generally less attrition in wounds from the air gun, owing to its propelling force being weak, than in those produced by gunpowder.

*Wounds produced by Gunpowder.*—The effect produced by balls or other hard substances when set in motion by the explosive force of gunpowder, is familiar to all. That the common wadding of a gun may produce very dangerous and even fatal effects, is however not so generally known. As the knowledge of this fact may be useful, and, as it has a practical bearing on medical jurisprudence, we shall mention two interesting cases related by M. Dupuytren: "Some years ago, a person, armed with a gun, while quarrelling with another man, fired upon him. The distance was short, and a wound was inflicted upon the abdomen;—this cavity was penetrated, and the small intestine wounded. As the patient died,—the case became the subject of judicial investigation. M. Dupuytren was called upon to declare the cause of death. The prisoner insisted that the gun was simply charged with powder, and that his intention was only to frighten, and not to kill his adversary. His statement was true; on examination after death, no ball was found in the wound.

As much depends upon the swiftness and force of propulsion as upon the density of the moving body. Thus M. Paillard and Marx have determined, by experiment, that a ball of soft, chewed, and wet paper, is able to perforate a plank of considerable thickness: the same thing was observed of balls of wax, dough of bread, and the end of a candle;—grains of powder, loose in the barrel of a gun, may be driven into the body to a considerable distance. We shall adduce one more instances of death by the wadding of a gun: "During the celebration of July, 1830, a merchant's clerk armed himself with a gun loaded merely with powder and wad. He discharged it about the distance of six feet upon a boy, with the intention merely of frightening him, without suspecting the occurrence of any accident; his error however was soon perceived; and the boy seriously wounded, was brought to the hotel dieu. There was a wound, the size of a five franc piece, upon the right flank, on a level with the crest of the ileum, five or six lines behind the anterior superior spinous process; the skin and superficial part of the muscles were taken away, as with a punch (emporte piece;) the bottom of the wound presented a black and bruised appearance. The iliac crest was uncovered to the extent of half an inch. There was some stupor, and very little hemorrhage. Under the influence of appropriate dressings and general treatment, the eschar separated, and the peritoneum was seen naked. Nevertheless every thing seemed to promise a favorable termination, when the patient having indulged too much in eating, the supperation was suppressed, the wound inflamed, aud chills and fever supervening; he died on the fifteenth day. On dissection, the peritoneum was observed to form a tumor, of the size and form of an egg, at the bottom of the wound—the illiac crest to the extent of an inch was denuded, abscesses formed around the wound, and the periosteum of the illiac fossa was detached, and elevated by pus. The peritosteum of this region was the seat of intense inflammation—the pleura of the right side was inflamed, covered by false membranes, and its cavity filled by sero-purulent fluid. The right lung presented on its surface, and in its substance, a number of small abscesses—some were observed in the inferior lobe of the left lung; the other viscera were sound.

Under the head of physical effects of projectiles, M. Dupuytren gives us much that is curious and interesting. Our limits will not, however, permit us to give much upon the subject. In the effects of balls upon bone, it has not unfrequently been observed, that the bone has been perforated, or that the foreign body may have remained in its substance, without giving rise to fracture. Particularly is this the case in young subjects, where the bones are as yet somewhat soft and yielding. M. Dupuytren relates several cases of this kind, and several have been observed by M. Paillard and Marx: "Kinderman, aged twenty-five, soldier of the imperial guard, was wounded by a ball, which traversed the tibia in its superior third. After suppuration was established, several dead portions of bone were extracted—a probe could be passed entirely through the limb; there was no fracture; and the patient perfectly recovered." A case occured to M. Larrey, in which the arm was amputated at the shoulder joint, for a wound of this part inflicted by a ball; on dissection of the limb, it was discovered that the ball had passed through the head of the humerus without producing any fracture.

The modification which balls experience in their course when striking upon water, has often been observed. If the angle of incidence is very great, that of reflection must correspond to it, and accordingly we have a series of skipping or bounding of the ball, on the surface of the water. If the angle is not so great, it may be reflected to a considerable distance; and balls which have been fired upon the water from one bank, have struck persons on the opposite side. We here cite a case which will illustrate this position, and may teach us the necessity of caution in similar circumstances:—"Two boys were firing at fish, upon opposite sides of the river, their guns being loaded with shot. One of them perceiving a fish, fired at it—at the same moment his friend, placed on the other side of the river was struck in the eye by a shot. A small wound was perceived on the inferior eyelid, and an opening upon the globe of the eye, near the union of the transparent cornea with the sclerotic. The sight of the eye was lost—violent inflammation took place, and only terminated by the bursting of the organ and evacuation of the humors."

Several pages are dedicated to the treatment of wounds inflicted by fire-arms, and many interesting cases are detailed. Inasmuch as the treatment does not present any thing peculiar, (with the exception, perhaps, of using incisions, to anticipate the occurrence of strangulation of the part, much oftener than is practised by most English and American surgeons,) and our limits do not permit us to make extracts from the cases, we shall refer the reader to the work itself, and pass to the consideration of amputation—the concluding subject of the first volume.

This is one of the most important topics which can engage the attention of the surgeon, and the preservation of the life of the patient, often depends not only upon his correct, but *prompt* decision. This is particularly the case upon the battle field, where, surrounded by a multitude of cases which demand his care and attention, by the noise and tumult of battle, he has little time for deliberation, and must act quickly and energetically. Many circumstances are here to be considered, which have a most important influence over the welfare of the patient, and which seldom occur in private practice. Patients who have sustained injuries at home, and have recovered without amputation, would probably have died, had these occurred in time of war, and had they been subjected to all the fatigue, care, pain, anxiety, and want of sufficient attention, which they would often have been obliged to experience. Indeed, the circumstances accompanying the injury are often as important to be investigated, as the nature of the wound itself. With respect to some wounds, there can be no doubt, all judicious surgeons agreeing, as to the necessity of amputation. Such, for instance, are cases in which a limb is carried off by a cannon-ball, those in which a large joint has been disorganized, and where the principal vessels and nerves of a limb have been destroyed, with fracture of the bone, etc. But although some cases occur in which no doubt can exist, there are others which demand, as we have already said, all the judgment, and profound reflection, of the practitioner. Some cases of compound fracture, are of this nature. When the parts are much contused, and there are many splinters of bone scattered in the soft part, and particularly if there is a lesion of the principal artery or nerve, the indication for amputation is evident. If, on the contrary, the splinters of bone are few, and there is little injury of the soft parts, the limb can generally be preserved. In compound fractures, occuring in battle, most surgeons are in favor of amputation. Still, notwithstanding this general precept, there are exceptions. Cases of cure are recorded, where the injury has been exceedingly great, the patients refusing to submit to amputation. Cases of this kind are recorded by almost every writer on surgery. These, however, do not affect the general rule. "Some cases are so difficult of decision," says M. Dupuytren, "that it would be necessary for God to send angels upon the earth, to practise surgery, and decide rightly these doubtful cases." The division of the principal nerve of a limb, never can, of itself demand amputation, except when tetanus ensues, and will not yield to ordinary means. "Amputation then presents a resource sometimes useful, but most generally of little avail." "Where there is lesion of both the main artery and nerve of a limb, amputation, is not absolutely called for; however, it cannot be denied that the chances of cure are small, inasmuch as the limb is deprived of its elements of nutrition, for a time at least, and of its sensibility and movements forever. If the soft parts are much injured, and especially if there be fracture of the bone, amputation becomes indispensable." The remaining observation of M. Dupuytren on this subject, are very interesting, but we must leave them, and pass to a subject, equally difficult, viz.—the time at which amputations should be performed. Formerly there was much difference of opinion on this subject, some surgeons contending for immediate amputation, others for postponing it until the symptoms of inflammation, etc., had disappeared. Among the advocates of this latter opinion, was M. Faure, to whom the prize dissertation on the subject was adjudged; but the experience of almost all modern surgeons is in favor of immediate amputation in cases of gunshot wounds. By immediate, however, it is not meant that the operation should be performed instantaneously, or before the first symptoms of depression, or prostration, shall have subsided. The shock of amputation, added to that which the system has already sustained, cannot fail to be injurious, and often fatal. We should endeavor to select a period intermediate to that of depression and re-action, so that we may not run the risk of extinguishing the vital spark, or, by delaying too long, induce the inflammatory state of the system, by the excitement of the operation. When the warmth has returned to the surface, the pulse has regained some force, the intellectual faculties are in operation, and the courage of the patient has returned, we may then operate. By so doing we save the patient from the long and tedious suffering, and risk of life, to which he would have been subjected, should we have previously determined to run the risk of secondary amputation. According to M. Dupuytren, and most surgeons, this period generally extends from one to six or eight hours, depending, of course, upon the constitution of the individual, and the nature of the injury. With regard to the mode of amputation, and the dressing to be employed, M. Dupuytren adopts the usual plan, and prefers union by first intention. We have endeavored, thus far, to give the reader a general idea of the nature of the work, and if it should appear that some parts have not been sufficiently dwelt upon, it was because they presented nothing new, or with which the profession have not long been familiar. We have sought to select such parts, as it was hoped, might prove interesting. In the next volume, wounds of the different regions will be considered.—*N. A. Archieves, May*, 1835.

C. E. I.

2. *On Scrofula.* By James Eager, M. D.—Dr. Eager has published a somewhat elaborate paper on the nature and treatment of a disease, which proves a great scourge in this country. The author was a house surgeon in some of the Parisian hospitals, and had good opportunities of witnessing the effects of various modes of treatment, and especially the treatment of iodine. On the nature and causes of this too well known disease, we need not dwell. The exhibition of iodine on a large scale at the Hospital of St. Louis, in Paris, and the wonderful cures which are said to be performed there, engage our author's chief attention in this paper, and our's also.

"The use of iodine requires the greatest prudence. It has been administered internally both in simple form and combined with iron, mercury, and potass, in the form of pill or in solution. Externally, combined with lead under the form of ointment, or dissolved in water in its simple state, either in lotion, injection, or bath. All these means have had their respective advantages. They were consequently used on various occasions. Iodine taken internally constitutes the basis of the treatment where the stomach is not affected. The results of cutaneous absorption are too doubtful to admit of frictions as the principal form of administering iodine. Children take pills with much difficulty. Pure iodine may be given in the form of tincture diluted with water. Two reasons, however, prevent us from using the tincture at this hospital. In the first place the fear of mistakes in dropping, mistakes the more easily made when there are many children under treatment; and secondly our apprehensions lest the water of Arcueil (which is used exclusively in this establishment, and which contains a large quantity of calcareous salts,) may alter the nature of this medicine. The same objection holds good with regard to the æthereal tincture of iodine, the simple or ioduretted solution of hydriodate of potass, and the alcoholic æthereal tincture of ioduret of mercury. We use the solution of iodine in distilled water called by Lugol, 'Eau minerale iodee.' Each pint of water contains two grains of iodine and four grains of hydriodate of potass, the latter being added to render the former more soluble. We have not deemed it prudent to adopt the use of the solutions of different degrees of strength which Lugol employs; because in the treatment of children, particularly when they are numerous, these different proportions offer many inconveniences without any real advantage. It is much more simple to administer a solution that contains a fixed quantity of iodine, which may be prescribed at will in more or less large doses, than to have for each patient solutions that contain a variable quantity of iodine.

The dose varies with the age, the state of the digestive canal, its influence on the disease, always beginning with three ounces of the solution, which may be gradually augmented to twelve ounces in the day. This is the strongest dose we have given, viz. ℥vj. in the morning and ℥vj. in the evening. Each ounce contains the $\frac{1}{8}$ of a grain of iodine and $\frac{1}{4}$ of a grain of hydriodate of potass. In following this method we know the exact quantity prescribed. After the use of this proportion for some time, we thought to double the quantity of iodine for each pint, but were soon obliged to abandon it in consequence of the disagreeable taste of the medicine, and a sensation of heat which it occasioned in the throat. The solution may be sweetened immediately before use with sirup of gum. If mixed with this sirup long before hand, the iodine becomes decomposed, and the solution loses both its color and taste. In order to preserve it long fit for use, it should be kept in bottles well corked and opened as seldom as possible. The smallest dose to begin with is ʒj. morning and evening, which may be increased to ℥vj. each time as beforementioned. This dose is even stronger than that Lugol recommends for adults. When no accidents occur to contra-indicate its use, we continue this solution during four or five weeks, then we stop it, and give a purge of sulphate of soda or magnesia. The purgative is repeated two or three times before we return to the use of the solution. The suspension continues generally fifteen or twenty days. We then resume, and continue its use a month, then stop it again to give the purgative as before said. It sometimes happens that acute accidental diseases oblige us to discontinue the treatment for some time. Diarrhœa and emaciation, which have been so much dreaded, seldom occur; when they do, they always cease on stopping the medicine. I have seen but one case in which the patient became thin; all the other children, on the contrary, have become very fat. The appetite has increased, and their soft flesh become hard and colored. I have seen a case in which the use of iodine was followed by cardialgia, and this affection ceased on using ℥j. of bark once a day, without discontinuing the iodine; and after a short time we were able to give the medicine alone. It sometimes happens, though seldom, that the use of iodine ulcerates the mouth and affects the breath as in mercurial salivations. In some females, symptoms of cerebral congestion appear, such as epistaxis, which yielded to rational treatment. It is remarkable that these symptoms manifested themselves at the period of puberty, and consequently may be ascribed more rationally to a deviation of the menstrual flux, than to any other cause. Messrs. Gardner and Coindet attribute to a kind of iodine saturation, accidents which have been remarked, in some patients, such as fevers, palpitation, dry cough, tremblings, loss of strength, and swelling of the legs. At the Children's Hospital we have not remarked such cases. This may be owing to the precaution we use of purging the children from time to time. Moreover, these symptoms are extremely frequent in the third stage of phthisis in patients who never used iodine. The ioduret of iron may be given with good effect in this affection, more particularly in cases where the patients are very pale, and their flesh soft and flabby. This medicine may be given in larger doses than the hydriodate of potass. We began by half a grain in ℥j. of water morning and evening, and increase it half a grain every four days. It may be increased to ten grains a day; in higher doses it induces vomiting. The solution of ioduret of iron produces diarrhœa much more frequently than the iodine solution. Iodine frictions are very useful auxiliaries to its action internally. We rub the tumors with an ointment composed either of ioduret of lead, ioduret of mercury, or ioduret of potassium. It sometimes happens (as with other medicines in all diseases,) that the ointment first used brings the tumor to a certain degree of diminution, and then it remains stationary. In such cases it is useful to substitute one of the others, and by this means the absorbent system is kept in a state of salutary excitement. The ointment of ioduret of mercury is composed of ℥j. axunge, and ʒss. of ioduret of mercury, that of lead of ʒj. to ℥j. of axunge, and that of ointment of hydriodate of potass with iodine, of ℥j. hydriodate and twelve grains of pure iodine to each ounce of axunge. The ointments of ioduret of mercury and potass determine a pricking pain that continues fifteen minutes, that of lead does not produce this sensation although applied to ulcers, spread on linen or charpie. A child ought to be rubbed but once a day, and an adult twice, in consequence of the skin being less irritable, and absorption less active in the latter; each friction during five or six minutes; and the quantity of the ointment varies with the size of the tumor, the minimum being that of a small pear or nut. We have in no case relied on frictions as the sole means of cure. They have always been combined with iodine internally and in baths. It is a matter of perfect indifference to which of the ointments you give a preference. For injecting fistulous trajets we use a solution of twelve grains iodine and twenty-four hydriodate of potass in one pint of water."*

An iodine bath is used in the hospital; but Dr. E. should have given us English weights and measures, instead of litres, etc., which are unintelligible to most people on this side of the Channel.

"Sixty-seven female children have been treated with iodine for a period sufficiently long to enable us to determine its effects with precision. Their age varied from four to fifteen years, a little more than half were over ten years old. In all of them the disease was of long standing; one was in the hospital since 1822, two since 1826, six since 1828, ten since 1829, twenty-six since 1830, and thirty-two were admitted since 1831, either before or after the 1st April, at which period the treatment commenced. In all these children the

* Dublin Journal, July, 1834.

disease presented itself under different forms. Fifteen of the sixty-seven who were treated by iodine were discharged cured of the apparent symptoms of the disease; fourteen experienced considerable amendment, which announced a speedily approaching cure. In thirteen others, although the improvement was less evident, one could judge that the cure would take place at some not far distant period; five received but little benefit from it, and, in fine, twenty did not receive the slightest advantage from the use of this medicine. Among the children cured was one admitted in 1826, two in 1829, seven in 1830, and five in 1831. Among those who experienced considerable benefit, was one admitted in 1826, two in 1828, three in 1829, five in 1830, and three in 1831. Of the twenty who were not at all relieved, two were in hospital since 1828, four since 1829, seven since 1830, and the remaining seven since 1831. All these children, with a few solitary exceptions, were much more affected at the period they commenced the use of iodine, than at the time of their admission. The disease had increased with their sojourn of many months, and even years, in the wards."

Seventeen of the sixty-seven had glandular swellings. In only four of these did the tumors entirely disappear. In thirteen cases the iodine completely failed. When left to nature, the ulcers of scrofula take many months to heal; and even then leave ugly cicatrices. "On the contrary, when art interferes, the cure is obtained in fifteen days, or a month at the farthest." Our author concludes by averring that no other remedy hitherto used in scorfula has been so successful as iodine. The paper appears to be a translation from the French rather than an original essay; nevertheless it is well worthy of perusal by practitioners in this country.—*Medico-Chirurgical Review, October*, 1834.

3. *Nitrate of Silver in Epidemic Cholera.* By Charles Lever, M. D.—We should not have noticed this subject, were it merely as setting forth a remedy for a disease which lately frightened these isles from their propriety. But it is on another account, namely, the safety with which the nitrate of silver may be taken internally, and that in large doses. The name of the medicine, and its caustic effect on the external surface of the body, have checked its internal use; though the phenomena attendant on its application to sores and morbid structures generally, might have lessened the fears of practitioners rather than increased them. The nitrate of silver is applied to an irritable ulcer on the leg or other part, and the pain is very little, while the irritability is seen to be materially lessened in a short time afterwards. To give a still more striking example: the fauces are red and sub-inflamed—the passage of food gives much discomfort; and there is a constant secretion of viscid saliva from the parts, clearly the product of inflammatory action. A few applications by means of a sponge to the fauces relieve or cure all these symptoms. Now it is to be remembered that the membranes at the extremities of all tubes are much more sensitive than any other portion of the canals; and therefore, as we find that the fauces, the pharynx, etc. will easily bear a solution of nitrate of silver containing five or six grains to the ounce, why should we fear that the stomach itself would be unable to bear a similar medicine? We have exhibited the nitrate of silver to some hundreds of patients—often in the dose of four and six grains a day, and never saw any injurious consequences result. The remedy is exceedingly valuable in many affections of the stomach.

In our Liverpool contemporary of July last, Dr. Lever has stated that during extensive observation of cholera in the cholera hospitals of the north of Ireland, he was led to attribute the general failure of remedies to the great irritability of the stomach, which prevented the reception, or the retention of medicines. The first case which Dr. L. relates was one of the most hopeless and deplorable character. The face, neck, and chest were deeply livid—skin cold—fingers corrugated—hearing gone—voice inaudible—pulse imperceptible—thirst insatiable; but vomiting incessant, whenever any thing was taken into the stomach. In this forlorn condition, Dr. L. dissolved thirty grains of the nitrate of silver in three ounces of distilled water, which she swallowed at once. She lay quiet for six minutes, when she vomited up a small quantity of whitish turbid fluid, and then remained perfectly at rest for twenty minutes, when Dr. L. left her. In three hours he returned, and found that the patient had sunk into a kind of slumber, and had had no recurrence of vomiting. The other symptoms remained the same. She continued in this state till the next day, without sickness; but with intense heat in the stomach. She drank freely all the night of weak brandy and water, without rejecting any of it. Reaction took place, and she completely recovered. Dr. L. saw the patient for nineteen months afterwards, and she never evinced any symptom of gastritis or other affection of the stomach. In the second case twenty grains of the nitrate were given in an ounce of distilled water. Most of it soon came up; but the vomiting ceased, and recovery took place. The usual dose, however, was ten grains to the ounce of distilled water. We think this document valuable, not only as respects the treatment of cholera; but as proving that the remedy, even in large doses, is unattended by danger.—*Ibid.*

4. *On Manual Assistance in Abortion.* By Mr. Wainwright, of Liverpool.—Mr. W. has published a paper on this subject in the 3d number of the Liverpool Medical Journal, which deserves attention. Mr. W. does not advocate indiscriminate interference in cases of abortion: because, under proper treatment, the majority of cases will require no manual assistance. He properly observes, also, that "until every hope is lost of preserving the ovum, or until the condition of the parent demands energetic assistance, the adoption of manual aid would be altogether unjustifiable." Mr. W. contends that, in all severe cases of abortion, the state of the os uteri should be ascertained, and that as early, and as effectually, as during actual labor. This examination is peremptorily required when the discharge goes on from day to day, confining the patient to bed, and blanching her countenance. The following appear to be the practical maxims laid down by our author.

"1st. In many instances, a *separation and partial expulsion of the ovum* will be found, and very slight assistance, by moving it from side to side, and gently drawing forwards, will at once remove the whole from the orifice, and the hæmorrhage will immediately cease. Case No. 2 offers an example of what may be experienced under those circumstances. The assistance required in this case was certainly not slight, nor easily afforded, yet the end was fully attained, and no mischief occasioned. It will be observed that I found it requisite to steady the uterus whilst I introduced the fingers through the orifice, and that this was effected by the left hand applied above the os pubis. I must add, however, that I do not think this plan will be effectual in every case, as in some individuals I have found it almost impossible to overcome the action of the abdominal muscles, especially of the *recti*. These seem to act, in some cases, almost involuntarily, when the hand is applied to the abdomen, whilst in others, the hand may be forced with little difficulty into the pelvis. The vagina, too, will offer some difficulty occasionally, and doubtless more when the patient has never borne children. It is unnecessary, however, to dwell upon this point, as it must be evident that unless the hand be allowed some degree of liberty in its movements, the operation in question cannot be effected.

2dly. The next two states I would allude to, are, first, where the *os uteri is found dilated, or dilatable, and a body is found projecting above;* and secondly, where the *os uteri is found dilating, and the membranes projecting*: especially during the continuance of a pain. These cases I believe to be, in many instances, the same, only in a different stage. I say, in many instances, because I am quite aware how often the tender membranes give away, allowing part of the conception to be expelled, whilst the residue remains. I class them together, however, because the same treatment is often required. Should the symptoms not be particularly urgent, and especially if the pains be frequent, it may be well to wait a little, that, in the first case, the contents may be propelled somewhat lower, and that in both, if possible, the natural efforts may accomplish the expulsion. Under other circumstances, however,—as Cases No. 3 and 4 will show,—manual assistance may be effectively and safely given.

3dly. It will sometimes be found, on enquiry, that a *fœtus has been expelled, but no secundines have been discovered.* Of course a most diligent search will be made for these, as it is very possible they may have been overlooked, or mistaken for a coagulum. Should the search however, be in vain, I could not acquit the practitioner of neglect, did he omit a vaginal examination, and were his patient afterwards to suffer from any of the effects of retained placenta. The decidua will often be found hanging at the os uteri; or if the pregnancy be rather more advanced, say about four months, it is not improbable that the funis,—or some apology for it, or a shred of membrane, or something,—may be suspended at the vagina, or at the os internum, and then the existence of something more within the uterus may well be inferred. Case No. 1 was of this description, and the treatment I adopted then I firmly believe to be the best that could be employed. The patient recovered extremely well. Some time had elapsed before I arrived, and I doubt not this explains the contracted state of the parts, and the difficulty I had in extracting the portion which remained. I think, therefore, as little time should be lost as possible; and that a gentle and cautious effort should be made to dilate the os uteri, and to extract that which remains. In the latter periods, at least, it may be remembered that the orifice has once been dilated to admit the passage of the fœtus, and surely little injury can be inflicted by distending it to the same extent, and often little more will be required. Here I would remark, that much misapprehension has been caused by this observation, 'that as before the sixth month the *hand* cannot be introduced into the uterus, therefore no manual aid can be given before that period.' Now the truth is, that in the earlier months it is absurd to think even of introducing the hand. One, two, or three fingers are all that are required, and that are almost ever spoken of. Case No. 6 exhibits the fatal effects of allowing the placenta to remain. It is true, that in this case the first gush of blood might be sufficient to account for all the consequences, yet the subsequent losses must have contributed to the fatal result.

4thly. At other times, however, whether a portion of the embryo be expelled or not, *a rigid state of the os uteri* will be found, absolutely defying the most resolute to overcome it. One author, I find, confesses he could not succeed, though 'he tried with all his might, and repeated his efforts ten times!' This, it may be presumed, would oftener be found in first pregnancies, and here, I hope, no one will follow the example of this courageous practitioner. I may add, for the credit of modern obstetricy, it was not a modern accoucheur. This, then, is the case of all others for the tampon. I have never met with this case, and I must rely, therefore, on the testimony of others. It is on this also that I rely, when I suggest, during the progress of such a case, the propriety of occasionally ascertaining the state of the os uteri. It may not be dilated at one visit, whilst it may be amply so at the next, and might then admit of manual aid.

Lamotte was called at two in the morning to an abortion of five or six months, from accident, attended with severe flooding. The os uteri was very rigid, but at six it was sufficiently dilated, and he effected the delivery. Giffard relates the case of a woman at about her sixth month, who had flooded for two or three days before he saw her. On the first day of his visiting her, he found 'the os internum not dilated enough to admit the end of one finger, and not easily to be dilated; on the second day she grew weaker, but still he could not dilate the orifice, but on the third day it was in a different state, and he quickly delivered her, when the hæmorrhage immediately stopped.' The same author relates an interesting case (No. 153,) advanced only to the ninth or tenth week. 'The woman had had hæmorrhage for some days, so that she was very much sunk, and had fainted several times. The os internum was very narrow, not allowing one finger to pass up; yet with the end of one finger he felt a soft substance within, lying at the mouth of the womb. She had much pain in the back. Three hours after, the orifice admitted one finger, which he passed directly through, and, bending its extremity, hooked out the contents, when all pain and flooding ceased.'

The above quotations, I think, will be admitted as warranting the practice I have suggested under this head, and may also be taken as strongly illustrative of the general question.

5thly. The above cases I supposed complicated with hæmorrhage. There is, however, another class of cases, unaccompanied with this alarming symptom, in which I believe manual aid may most advantageously be afforded. I allude to those in which the fœtus has been expelled with *little or no flooding, but where the secundines* (*decidua or placenta*) *remain.* Cases No. 5 and 7 are instructive cases in point. The treatment adopted by Mr. Park fully shows the practice usually employed, and this, with his observations attached, may, in the opinion of many, fully authorize its adoption in future. Few,

I am sure, hold that excellent surgeon in higher estimation than I do, yet I cannot withhold expressing what I fully believe experience will justify me in stating. It is universally acknowledged, amongst writers, that a woman is unsafe until the placenta is expelled, and who has not witnessed the uneasiness both of patient and friends until this is accomplished, proving a general consent to the same fact? In case No. 5, I left it, and the consequence was that it was detained two months, during which time the patient was never well; and at length it was expelled with a fearful hæmorrhage. In the sixth case it was left, and for six days both medical attendants, patient, and friends were in constant doubt of something going wrong, but to our relief it was then expelled, a disgusting mass. These, however, I consider good escapes, rather than proofs of good treatment, because I find that reports of more serious consequences abound in the writings of those who have enjoyed the widest fields for observation. Dr. Burns says, p. 297,

'If the gestation has not advanced far, the placenta may be expelled in small portions for many months, but the patient is all the while languid, hysterical, and subject to irregularities of the menstrua, and very often to obstruction. But more frequently the symptoms are very acute. We have loss of appetite, prostration of strength, tumid or tender belly, frequent small pulse, hot skin, and various hysterical symptoms. The discharge from the vagina is abominably fetid, and hæmorrhage sometimes occurs to a violent degree.'

Mr. Ingleby's observations are to the same purport, and he relates a fatal case from the placenta being retained. (p 118.)

Smellie gives a case from Mauriceau, vol. 2, p. 171, strongly proving the same point.

A woman miscarried at the fourth month, and the placenta was left entire. The midwife said the womb had closed, and she could not extract it. He saw her on the third day, and found her statement correct. He could not remove it. She was at the brink of the grave, from 'a prodigious flooding.' The placenta became putrid, and a most violent fever ensued, 'accompanied with exacerbations two or three times a day, and with faintings and other symptoms usual on such occasions.' The placenta was at length expelled, and the woman recovered.

Smellie mentions another example, which happened in the practice of one of his pupils. (Vol. 2, p. 167.)

The placenta was retained after an abortion at the fifth month. The patient was subject to constant draining for three months, until she became pale and emaciated. At this time he visited her. The os internum admitted two fingers, with which he was enabled to separate a hard round substance from its attachment to the uterus, but could not bring it away. He then used a blunt hook, and removed it in three portions. The discharge ceased, the woman recovered, and afterwards bore children.

The above cases clearly prove the great importance, and, in my humble opinion, the absolute necessity, of removing, in all possible cases, the whole of the uterine contents; and here I must especially urge the removal being accomplished as quickly as possible. It is a remarkable, but a well-established fact, that the uterus and its orifice become almost permanently contracted very shortly after even a partial expulsion of its contents. Cases No. 1 and 7 in this paper show the strong tendency to this contraction, but other cases prove it completely. MAURICEAU mentions a person who miscarried at the sixth month; part of the after-birth remained, and before he arrived the os uteri was so closed he could not effect its removal. Dr. DEWEES dwells particularly upon this fact. (P. 414.)

I feel it, however, a duty to state, that Dr. DENMAN, who appears to be the oracle of British accoucheurs, and in many respects most deservedly so, does not advocate the removal of the placenta when retained after abortion. His concluding remark is as follows. (4to ed. p. 490.)

'At all events, much less mischief may be expected from the retention of a putrid placenta at this time, than from attempts to force it away by the medicines usually given for that purpose, or by manual assistance.'

I would simply beg to make this observation. I can find no argument to justify the use of 'force' in this case, but between the mischief arising from a judicious endeavor to remove a retained placenta, and that arising from a putrefying substance within the uterus, an organ possessing sympathies so numerous and so important, I leave the reader to decide."

The cases themselves we are unable to insert, and they cannot be abbreviated.—*Ibid.*

5. *Observations on Catarrhal and Catarrho-Rheumatic Ophthalmia.* By FRED. TYRRELL, Esq.—In our quarterly contemporary for July last, there is a good practical paper on the above subject, from the pen of a good practical surgeon. Mr. Tyrrell remarks, that during the concluding part of last winter, and up to July of the present year, catarrhal ophthalmia has been more prevalent than he ever knew it to be. The disease was often so severe as to affect the sclerotic as well as the conjunctiva. The following is Mr. T's. descriptionof the

CATHARRAL OPTHALMIA.

' It commences with stiffness and heaviness about the palpebræ, with slight irritation or itching of the ciliary margins, and a sensation as if some fine particles of dust had lodged on the surface of the globe; there is usually slight intolerance of light, with increased secretion of tears and agglutination of the lids during sleep. After a short time the patient experiences pain, which is increased by motion of the eyelids, or by exposure to bright light. These symptoms are aggravated about sunset.

On examining the eye, there is perceived in the early stage slight thickening and redness at the free margins of the lids, and probably some coagulated secretion collected at the inner canthus and about the cilia. The ocular portion of the conjunctiva exhibits a few tortuous vessels filled with red blood, whilst the palpebral portion is red, tumid and villous, like crimson velvet; and in the lower folds between the ocular and palpebral parts of the membrane a little whitish or slightly yellow and thick mucous secretion is lodged.

The inflammation, however soon spreads to the ocular conjunctiva, the vessels of which become extensively injected with red blood, and, on close inspection, are found to form a beautiful net-work. In severe cases, the conjunctiva is partially thickened and elevated by deposition of serum between it and the sclerotic, constituting a partial chemosis, and occasionally small spots of ecchymosis are perceived from the rupture of some minute vessels.

If the disease extends farther, it produces ulceration of the cornea.

This ophthalmic disease seldom exists without some symptoms of general catarrh, such as headache, sense of fulness about the frontal sinuses, sneezing, increased secretion from the nose, and some general febrile symptoms: the local and general diseases are usually reciprocal.

In young subjects the catarrhal-ophthalmia is often modified by a strumous diathesis, which occasions the local affection to be more severe, augments especially the intolerance of light and lachrymation, and disposes to the formation of pustules.

This disease may occur at any period of life. It is produced by a peculiar state of the atmosphere, during the continuance of which there is considerable difficulty in entirely removing the ophthalmia."*

The treatment must be modified by the severity of the symptoms, the extent of the constitutional disturbance, and the condition of the general power.

"When there is simply affection of the palpebral conjunctiva with morbid mucous secretion and little general disturbance, and sufficient constitutional power, I prescribe a brisk aperient of calomel and colocynth, or calomel and rhubarb, or jalap, and afterwards a small quantity of some of the preparations of mercury with antimony, or with Dover's powder at night, and a mild saline aperient in the morning, also some mild diet, principally vegetable and farinaceous, with quiet, and the avoidance of cold or damp, or of bright light.

Locally, I employ merely tepid water, to cleanse the eyes; or warm water to foment them when uneasy, and some simple ointment, which should be smeared upon the lids and eyelashes before the patient goes to sleep.

When the local disease is acute and the constitutional affection considerable, general or local bloodletting must be resorted to; the former being only necessary in persons of naturally full habit or subject to inflammatory diseases; and after bloodletting the treatment above described will be applicable.

In a few days the mild or acute form, if treated as I have described, passes into the chronic stage, which is denoted by a general mitigation of symptoms, but more especially by an alteration in the mucous secretion, which becomes thin and pale, and by the change in color of the conjunctiva, which loses its brilliaht aspect, and presents a more pale and lax appearance; at the same time it is found that the general power is failing under the continuance of the constitutional affection; the treatment must therefore be changed. Instead of exciting the secretions, they must now be kept as near as possible in their natural state, and a more generous diet must be allowed, but still the patient must abstain from such food as may tend to excite vascular action. It is necessary, in persons of feeble power, to give, in addition, some medicinal stimulus, as some of the preparations of bark, mineral acid, or ammonia.

To subdue the remains of the local disease, a slightly astringent lotion must be substituted for the tepid water, as a solution of the acetate of lead, half a grain, or a grain, to the ounce: or a solution

*Med. Quart. Rev. No. IV. p. 416.

of alum, a grain or two grains to the ounce; the sulphate or acetate of zinc, a quarter or half a grain to the ounce, or nitrate of silver in the same proportion. Also a stimulating ointment must be applied instead of the milder form, as the dilute citron ointment composed of half a scruple or a scruple of the unguentum hydrargyri nitratis, to two drachms of unguentum cetacei, or a scruple of the unguentum hydrargyri nitrico-oxydi to a drachm of the unguentum cetacei."

Where a strumous diathesis prevails, and where, as was observed, there is much intolerentio lucis, counter irritation by blisters is necessary. The intolerance exists both in the acute and chronic form, which renders it difficult to distinguish between them, though it is necessary to discriminate in the treatment. Mr. T. is convinced that more benefit results from repeated blisters, than from keeping one open. Cold applications are injurious to the eyes of strumous children. He uses tepid water in the acute stage, and trusts to general remedies in the chronic. We now proceed to the

### Catarrho-Rheumatic Ophthalmia.

"This affection commences with symptoms similar to those of the catarrhal, but with more early suffering: for in addition to the symptoms of the catarrhal disease, the patient experiences a constant dull aching pain in the globe of the eye, and in the temple and eye-brow: the globe feels tense, and as if it had been bruised; and these pains and sensations become so much augmented towards evening as to prevent sleep. The accession of pain is accompanied with intolerance of light and a feeling of dryness of the conjunctival surface, so that the motions of the eyelid upon the eye produce excessive pain; but occasional gushes of tears occur, which for a few moments produce some relief; while the symptoms continue thus acute, the globe of the eye, the temple and eye-brow, are extremely tender to the touch.

In the intervals between the severe attacks, when there is but slight intolerance of light, vision is frequently indistinct, and occasionally small black spots or muscæ are perceived by the patient.

On superficial inspection of the eye, the appearances denoting catarrhal-ophthalmia of an acute kind are apparent; such as the red palpebral margin, the thick mucous secretion, the thickened and villous state of the palpebral conjunctiva, with the tortuous vessels and vascular net-work on the ocular portion of the membrane; but an attentive examination soon discovers that the color of the ocular conjunctiva is more uniform and deeper than in the simple catarrhal disease: and this upon further observation is found to depend upon the existence of another set of minute vessels filled with red blood, which can be traced in the intervals between the tortuous vessels of the conjunctiva as passing beneath them in a straight course from the margin of the cornea towards the orbit; these vessels are situated in the sclerotic coat; they are occasionally found more abundant in one part than another; as, for example, more upon the nasal than the temporal side of the cornea, and of course augmenting the depth of color in such particular situations: it requires, therefore, a careful examination of the whole ocular surface to detect them.

When the vision is affected, being cloudy or troubled with muscæ, some change is perceptible in the iris; its pupillary aperture is contracted, its brilliancy is diminished, or its color slightly altered; which circumstances are best detected by comparing the iris of the affected eye with that of the healthy organ: the motions of the affected iris also are sluggish. The iris suffers in consequence of inflammation extending to it from the sclerotic.

Sometimes a partial or complete ash-colored line is seen between the cornea and vessels injected with red blood, most frequently the line forms a complete circle around the cornea; but I have seen it merely occupying a portion of the circumferance of the cornea, being apparent at its temporal and nasal margins, and deficient superiorly and inferiorly. This appearance is considered by the continental and also many of our own ophthalmic surgeons, as a diagnostic of rheumatic inflammation. Such opinion, however, I consider to be erroneous, for I have seen a similar line present in iritis, simple sclerotitis, and choroiditis. I believe it results from the mode of junction between the sclerotic and cornea occurring when any of the diseases alluded to are present, and seen most distinctly when the junction of the two structures is very oblique, but not at all evident when the textures are joined with little or no obliquity. Now and then the margin of sclerotic overlaps or joins obliquely the circumference of the cornea, more in one part than another, and this I have observed most frequently on the temporal and nasal sides, so as to make the transverse diameter of the cornea less than the perpendicular diameter. When such formation exists and inflammation affecting the sclerotic occurs, the ash-colored line is apparent only towards the nose and temple."

The exciting causes are cold and moisture; and Mr. T. suspects that the simple catarrhal affection is often converted into the rheumatic, by the injudicious application of cold lotions.

### Treatment.

When inflammation preponderates in the conjunctiva, and the sclerotic is but slightly affected, leeches must be applied, and brisk purgatives given. For a few days, Mr. T. employs the same means as in the simple catarrhal affection, excepting that, when the circumorbitar pains are severe, he directs blisters, or a few grains of mercurial ointment, with opium, to be rubbed on the temple and forehead at night. As soon as the severity of the conjunctival affection has subsided, he prescribes a more nutricious diet, and small doses of cinchona and soda, sarsaparilla and lime-water, or some mild tonic with an alkali. Locally, warm water is occasionally used to cleanse the eye; "but the patient must carefully wipe it, and not allow the surface of the lids to remain moist, as I am confident it augments the mischief." The repetition of blisters is serviceable where there is much intolerentia lucis, or continual circumorbitar pains. When irritability, with little vascularity, remains, a drop or two of vinum opii once or twice in the twenty-four hours, dropped into the eye, is useful.

"If the catarrhal affection of the conjunctiva be slight, and the inflammation of the sclerotic severe, without, however, much affection of the iris, local bloodletting is rarely necessary. The patient is at first to be freely purged by some drastic medicine, after which a full dose of colchicum, with a narcotic, may be given, especially when there is any marked rheumatic diathesis: the best period for administering this dose is in the evening, a short time before the usual period of exacerbation. I have known it to subdue altogether the sclerotic inflammation, leaving but a slight catarrhal ophthalmia If the first dose appears to alleviate the disease, it may be repeated every six or eight hours for three or four times; but I have never found any benefit from a longer continuance of this remedy, nor have I seen much advantage from it when it has occasioned disturbance of the stomach or bowels. I consider its beneficial action very uncertain.

Otherwise, I prescribe according to the state of general power, which it is of the utmost importance to observe, in order to treat successfully. Very often, when the local disease appears acute, the general power is extremely feeble; or, on the contrary, when the local disease is slight, the constitutional power is good; but occasionally, the general action is energetic when the local disease is severe. When the action of the heart and arteries evinces deficient power, although the local symptoms and appearances may indicate an acute form of disease, any active depletion likely to affect the system ought not to be adopted; blistering is the best means of relieving the local congestion; besides which, medicines should be given to correct any error in the principal secretions, especially those of the bowels, skin, and kidneys: the two latter I find most frequently to be deranged. Antimonials with salines, are therefore most serviceable after the bowels have been acted upon. The patient must be allowed a good nutricious diet, and even a small quantity of stimulus, if much addicted previously to its use.

As soon as the secretions are in a healthy condition, tonics should be exhibited, and if the general power is good I prescribe five grains of the pulvis cinchonæ, and an equal quantity of the sodæ subcarbonas exsiccata every four or six hours; and I know of no remedy so generally serviceable in the state of the disease above described. I am indebted to Mr. Wardrop for the suggestion of this medicine. I have frequently known it succeed in relieving inflammation of the sclerotic, when the larger doses of the same preparation have been taken some time without benefit. Should this remedy fail, I give the sarsaparilla with lime water, but with either medicine it is necessary to administer an occasional purgative, with or without some mercurial, according to the state of the biliary secretions.

When the patient's power is feeble, I usually commence with the sulphate of quinine, as a tonic, in doses of three or four grains every four or six hours; and if there is a disposition to inordinate cutaneous secretion, I prescribe, in addition, twenty or thirty minims of the dilute sulphuric acid. Under these circumstances, also, a generous diet is requisite, and a moderate quantity of any stimulous to which the patient may have been accustomed. I have lately seen many cases in which the catarrho-rheumatic disease had continued, (principally, however, of a rheumatic character,) for six or seven weeks, or more; a depletory treatment having been persisted in, and in which a cure has been effected in as many days after the tonic plan has been adopted.

It sometimes happens that the disease has extended from the sclerotic to the iris before the patient applies for relief, or that the affection being treated under the supposition that it is merely a conjunctival inflammation, the remedies employed have not been sufficient to prevent the extension of the disease to the iris. Inflammation of the iris is indicated by a loss of brilliancy, an alteration in color, a thickening of the pupillary margin, and a contracted and sometimes irregular condition of the pupillary aperture. When the iris is but slightly diseased, there is no occasion to deviate from the treatment which I have just described; but if the inflammation of this texture is decidedly developed, it is absolutely necessary to resort to mercurial treatment to stop its progress, otherwise the plan of depletion which I direct in the early stage of catarhal or simple catarrho-rheumatic ophthalmia, may not arrest the inflammatory action in time to prevent deposit of lymph in the texture of the iris and in its pupillary margin, by which the pupil may be rendered permanently irregular, or its margin become in part adherent to the anterior capsule of the lens, and the transparency of the capsule itself be materially injured.

The exhibition of mercury must be carefully and guardedly resorted to, especially when the constitution is feeble, which I have observed it most frequently to be in persons suffering from this disease; nevertheless I deem the use of the remedy of the greatest importance, if given in small doses, at short intervals, whilst the patient is allowed a nutricious diet, and the secretions kept in good order; the surgeon may, by keeping a careful watch, always stop the exhibition of the medicine in time to prevent any mischief.

At the same time that mercury is given, the extract of belladonna should be applied to the eye-brow night and morning, to keep the pupil in a dilated state, and prevent any adhesions taking place between the pupillary margin of the iris and the anterior capsule of the lens, with a contracted pupil; or to aid in the separation of such adhesions, should they have been formed previously.

The mercurial treatment must be stopped as soon as the inflammation of the iris subsides, whether the system be affected by the remedy or not. I have seldom had occasion to give mercury so as to produce any marked effect on the system in these cases, and have always found that when the remedy has by accident, or inattention, created much general disturbance, the cure of the remaining disease in the sclerotic has been more difficult. The disease in the iris being subdued, the plan I have recommended for relieving the inflammation of the sclerotic should be immediately adopted.

In several instances which have come under my observation during the last few months, although the general and local diseases have not been very severe, the patients have experienced excessive debility, and have not recovered until they have had the benefit of change of air for a short time.

I have been desirous to shew, in the preceding observations, that it is of much importance, by careful examination of the organ, and attention to symptoms, to ascertain the extent of disease before treatment be commenced, and that the treatment must be varied according to the extent and severity of the inflammation; but especially to point out the existence of apparently severe local inflammation, with a feeble state of the constitution, which I have observed frequently during the late prevalence of these diseases; for I am satisfied that inattention to this circumstance has been the principal cause of want of success in the management of most of the cases which I have seen in consultation."

We have thus laid the whole substance of Mr. Tyrrell's paper before our readers, as it will prove serviceable to the practitioner in the daily rounds of his avocation.—*Ibid.*

6 *Lepra Vulgaris, invariably treated with success*, by Edward Beck, M. D. of Ipswich, Eng.—The object of Dr. Beck is to display the obstinacy of lepra and psoriasis—their indisposition to yield to the treatment usually recommended—and the superior efficacy of the plan which he proposes and pursues. Without further preface, we shall state the plan in question.

"When the disease is of considerable standing and attended with much pain, soreness, and inflammation of any one part, a preparatory plan of treatment will be required, which is to be principally antiphlogistic.

I have generally recommended abstinence from wine, and prescribed a few doses of aperient medicine; equal parts of Pil. Rhei Comp., and Extr. Colocynth Comp. with or without two or three grains of Pil. Hydr. answer the purpose well. The Sulph. Precip., in combination with Sod. Subcarb. Excis. in doses of half a drachm of the former to five grains of the latter, two or three times in the day, so as to act freely on the bowels, has a beneficial influence in reducing the irritation. The Liq. Plumb. Subacet. Dilut., or a lotion containing the Plumb. Subacet. in combination with Zinci Sulphas, I have found the best local applications, during the very irritable state of the disease: two scruples of the former, with half a drachm of the latter, to a pint of distilled water."

The preparatory plan, therefore, consists of active aperients for some days—the application of the lotion that is found to suit best—and, when much local irritation exists, the use of the powder of sulpher and soda during three or four times a day. Sometimes there is no necessity for a preparatory plan of this description. As soon as the irritation and inflammation of the parts are moderated, the specific treatment is required.

"The specific plan consists in applying the following linimen[illegible] the parts affected:—

℞.—Picis Liquid.
Sulphuris.
Adipis Præpar. sing. unciam misce.

The following pills are at the same time to be taken regularly:—

℞.—Picis Liquidæ unciam dimidiam.
Flor. Tritici q. s. misce. et divide in Pilul. sing. gr. v.—
iii. ad vi. ter in die sumantur.

Where the irritation is very considerable, a milder liniment may first be had recourse to, as the following:—

℞.—Picis Liquidæ.
Sulphuris singul. unciam dimidiam.
Adipis Præparat. uncium. misce.

This preparation, should it irritate very much, may be gently rubbed on the parts affected with the leprous eruption at night, allowed to remain on a few minutes only, and then wiped off with soft linen. The application usually increases the pain for a few minutes, but afterwards the parts become much more comfortable to the patient's feelings. In three or four days the former liniment can be borne and will be required; and it may be allowed to remain on the part all night, and be washed off in the morning, or it may be applied and remain on it for days together, for it is better that the part should not be frequently washed, as the local irritation is considerably increased thereby. Where a large surface, a limb for instance, is covered with the leprous eruption, it may be gently rubbed well over with the liniment, and the part covered with linen (in the form of a stocking if it be the leg, or a glove if it be the fore-arm, or back of the hand.) Every second or third day will suffice to apply the liniment afresh. This is a very efficacious mode of applying the liniment, but is adapted only for those in the lower class of society, who might have no objection to the scent of the ointment. Among the upper class of society, this plan might be seriously objected to, in which case the application should be used at night only, and washed off in the morning. But where single patches are situated, some of the ointment should be applied thickly to them, and two or three pieces of lint confined over it, with strips of adhesive plaster—this may be allowed to remain for days together, and no scent can escape if the lint be well confined. Most of the small leprous spots, which are not of long duration, will disappear under the general plan of treatment, and only an occasional large patch will require this attention for a short time.

As the pills sometimes occasion headache and disagree with the stomach at first, they may be commenced with by taking two, three times in the day, and very gradually increasing to six, three times in the day, which will be found an efficient dose, and as much as most stomachs will well bear. The torpid state of the bowels that almost invariably accompanies lepra, usually gives way to the use of the pills.

Where the disease is of short duration, and is not confluent, the preparatory plan of treatment may, in a great measure be dispensed with, both locally and generally.

A single dose of the pills at night, followed in the morning by a teaspoonful or two of calcined magnesia, or half an ounce of the sulphate, will be found sufficient; but, without some preparation for the pilulæ picis, they will be found to occasion nausea and headache, at which the patient, taking an early disgust, will with difficulty be persuaded to resume the use of them. In all cases, where the local irritation is considerable, the stronger form of the ointment may, from the first, be had recourse to. After the patient is able to take a full dose of the pills, the use of aperients may, in almost every instance, be discontinued, for the pil. picis alone regularly and sufficiently act upon the bowels.

I particularly wish to point out the necessity, where much local irritation is present, of using the milder Ung. Picis Comp., and that it remain on only a minute or two. Without this precaution, the patient may take a prejudice against the application, from the increase of pain it produces, and discontinue it altogether."

Both pills and ointment are necessary to effect a cure, and their use should be continued for some time after the disappearance of the disease. Two months, or even more, will be required for the cure of severe or long-continued lepra.

Our author's treatment of psoriasis is put forward with less confidence than that of lepra. It consists of anti-phlogistic treatment while heat and irritation are considerable—aperients—after a few days the application of an ointment, consisting of half an ounce of camphor, and two ounces of cerat. cetacei—and the exhibition of sulphur and soda.

We have no space left for comments, which we leave to the fancy, the judgment, or experience of our readers.—*Ibid.*

7. *Ophthalmia treated with lotions of Corrosive Sublimate.* By M. Bally.—M. Bally, in a paper published by him a few years ago, in the Gazette de Sante, strongly recommended the employment of a solution of oxymuriate of mercury in ophthalmia, whether of an acute or of a chronic character. The following two cases are adduced, as confirmatory of its good effects.

A woman was seized, after exposure to alternate heat and cold, with a violent inflammation of the conjunctiva of the left eye. The organ was exceedingly painful, and could not tolerate the impression of light —she felt as if sand had got in between the lids and eyeball, and the tears were hot and burning. The disease had lasted six days, when this patient consulted M. B., who ordered her to bathe the eye, a dozen times at least in the course of the day, with a collyrium, composed of four grains of the sublimate and four ounces of water. In the course of three days, the inflammation had almost entirely disappeared, and by the sixth the woman was pronounced cured.

In the second case, the ophthalmia had continued for eight weeks before the patient consulted M. B. He had been repeatedly leeched, blistered, and also well purged, but without much benefit. Both eyes were inflamed; and on the cornea of the right one there was a nebulous spot, of the size of a millet seed;—this eye was more painful than the other, and the sight was more imperfect. M. B. immediately ordered the use of the sublimate collyrium, and a blister on the nape of the neck. No change was made at any time in the treatment; by the 13th day, all inflammation had disappeared from both eyes; the speck however remained; but, fortunately, this also vanished under the application of "pommade de Janin," and the vision was completely restored. The author adds, that he could cite at least twenty other cases of ophthalmia, treated successfully by the same plan.—*Revue Medicale.—Ibid.*

8. *Best mode of treating Mammary Abscess.*—Mr. Robinson has communicated six cases of mammary abscess to the Medical Gazette,* for the purpose of displaying its history and treatment. We presume that the greater number of our readers are tolerably familiar with the former—that few need be informed of the occurrence of common abscess in the breast, as in any other organ or tissue—and that it is not necessary to describe at any length the mode in which obstruction of the lactiferous ducts may occasion inflammation, suppuration, ulceration of the integuments and fistulæ.

The question which has been disputed in reference to the surgical treatment of this complaint, is the propriety or otherwise of an early opening. Mr. Robinson's observations on this head are not very copious nor precise. They are as follow.

"The third case proves, that where the abscess is owing to the distention of the lactiferous tubes, the formation of fistulæ may sometimes be prevented.

The fourth case shews the result of opening by the lancet, and opening spontaneously. The former plan will generally be found the best.

* July 12, 1834.

especially in deep-seated abscess. In this case the opening made by nature was large, and the contents of the abscess were evacuated all at once, which is more favorable than usual; for generally the opening is no larger than a pin's head, and is slow in healing up, the skin being commonly much destroyed before it gives way. Even in this case that abscess was much longer in opening, and more painful, which burst spontaneously, than that which was opened by the lancet.

The fifth case more forcibly proves that the artificial opening is preferable to the opening by nature; for the first abscess being opened by the lancet, was healed in three days; whereas that which broke of itself was much slower in healing, and was slightly fistulous.

This also points out the necessity of watching these cases: the woman was desired to call on me the following day, instead of which she allowed three days to elapse; otherwise I believe the fistulæ would have been altogether prevented.

The sixth case shows this disease in its most distressing and tedious form: the breasts were permitted to become over-distended with milk; both breasts were inflamed throughout; numerous abscesses, implicating the lactiferous tubes, followed; and she suffered severely for ten weeks.

The two last cases prove the great advantage of pressure (after the inflammatory stage is gone off,) in obliterating fistulous canals—a plan which will in many instances prevent, if it does not altogether supersede, the necessity of extensively laying open the breast, a custom which used to be resorted to formerly for the cure of sinuses, and which is even in the present day, I believe, occasionally adopted."

These remarks embody little more than the usual recommendations and the common practice. The most annoying circumstance connected with abscess of the mammæ is the disposition to separate processes of suppuration, or, rather, to the formation and collection of matter in several portions of the same breast. They redound in some degree to the discredit of the surgeon, and form a serious source of distress to the patient.

It has been noticed in cases of diseased absorbent glands, and even in abscess of the cellular tissue, that suppuration does not always form in a single point, nor appear at the commencement inclosed in a single cavity. On the contrary, it has been found that it sometimes begins in several distinct portions of a gland, and that, some time elapses before one common abscess is produced. The practical rule has been deduced, to wait till such a collection has occurred before an artificial opening is resorted to. If the surgeon operates too early, he has to open in succession several abscesses instead of one.

Our own opinion is, that the fact should always be remembered by the surgeon, and that by it his practice should be mainly guided. Yet we think that too much stress has been laid upon the one, and that the other has been carried rather too far. In cases of venereal bubo, if the surgeon declines to open an abscess until a large suppurating cavity is formed, he will probably endanger the vitality of the integument, and certainly protract to an unnecessary extent the duration of the complaint. Our readers will find more detailed observations on the management of suppurating bubo in another place.

We suspect that the cases of mammary abscess and suppuration in a strumous or venereal gland are not altogether analogous. In the instance of the gland its whole texture is diseased, and will probably be invaded throughout by suppuration. But the mammæ is differently circumstanced. The lactiferous tubes are obstructed in one part—distention, inflammation, and suppuration in that part are the result,—and there appears to be no necessity why other portions of the gland should be involved. That they *are* so will probably be found to depend upon another cause. The gland is composed of numerous lobules, with adeps and cellular tissue interposed. Inflammation occurring in one part of the gland may readily extend along the cellular processes to another. The same thing would seem not unlikely to ensue, if matter were confined, or if it had not a free exit: and such is actually the case.

If these considerations are correct, an early opening of the mammary abscess is desirable. We are convinced, for our own part, that it is so. We have treated many cases of this complaint, and were at first annoyed, as all surgeons have been, by repeated suppurations and ulterior sinuses. It appeared to us that these depended on a simple opening not sufficiently evacuating the abscess. After some practical experiments we adopted the following plan with much success:

Having satisfied ourselves that suppuration was actually established, we made with a lancet as dependent an opening as circumstances would admit, a vertical puncture being generally preferable to a horizontal one. We then introduced a probe into the cavity of the abscess, and ascertained if any sinus extended from it to another portion of the breast—or if the point of the probe could be made to project beneath the skin, in a more dependent spot than the puncture. If either was the case we cut on the extremity of the instrument, made its point project through the incision, tied around it a narrow piece of tape, or a silk ligature, withdrew the probe, and thus established a small seton between the opening originally made and that obtained by cutting on the probe, at the most dependent portion of the abscess. If more than one sinus extended from the latter, or if its formation or situation was such that one dependent opening was insufficient to empty it completely and at once, another incision was made upon the probe in the requisite direction, and another seton passed in the same manner as the former. The whole should be done at one operation, and since we have adopted this method of management, we have not been annoyed by repetitions of abscesses or formations of sinuses. We have found it, in fact, a great improvement in the treatment of this painful and troublesome affection. The operation is rather more severe at the time, but when once performed it prevents the necessity in almost every instance, of others. A patient will go through one with fortitude, but her spirits are broken and her strength of mind is shaken by its frequent repetition.—*Ibid.*

9. *Observations on Erysipelas. By* Ephraim M'Dowel, M. D. *one of the Surgeons to the Richmond Hospital.*—This forms the leading article of our Dublin contemporary for October last. It appears that the disease was epidemic in the Dublin hospitals in the beginning of 1834, and very frequently fatal. It attacked all ages and both sexes indiscriminately—the sick and the healthy—while almost every kind of injury was followed by the disease. It occurred after leeching and bleeding, venereal ulcers, and even the application of liniments to the surface. Surgical operations were so generally succeeded by erysipelas, that they were performed as seldom as possible. Like the epidemic influenza, it was characterized by extreme prostration of strength, rendering depletion precarious, and often dangerous, even where there was strong local inflammation, and the constitution was sound. In the progress of the epidemic, there was great variety in the numbers attacked at different periods. Sometimes it would be nearly extinct in the hospital, and then unexpectedly burst forth with renewed force. It was most troublesome in Easterly winds.

"The different cases of this disease met with by the writer, were referable to one of three classes. In the first, occurring in healthy persons, and usually after injuries, there was much local and constitutional disturbance; the disease was often of the phlegmonoid form, but in other cases, the skin alone was acutely attacked. In the second class of cases, examples of superficial erysipelas, the redness was more diffused, frequently it was not very perceptible, and occasionally considerable patches of the skin were unaffected, passed over, as it were, by the disease; vesications occurred more frequently than in the more acute cases: this form attacked persons of weak constitution, or of unhealthy habits, in whom the biliary organs and stomach were deranged or diseased: the constitutional symptoms seldom ran high. Subcutaneous abscesses formed very insidiously, and frequently in considerable numbers, and, if not opened early, spread very quickly, death of the cellular membrane to a varied extent generally occured, undermining the skin, and protracting much the cure. It was often necessary to examine a limb most carefully to detect these purulent depots, the patient seldom being aware of their existence; the matter secreted was generally healthy pus. In one case of erysipelas of the head and face, succeeding ptyalism, slight pain, or rather uneasiness, had been complained of in the neck; there was no evidence of disease, but on examination after death, a very extensive *puriform* infiltration of the loose cellular tissue, about the trachea, thyroid body, and œsophagus, was found; it extended behind the pharynx nearly to the base of the cranium, and very probably was not merely an example of inflammation propagated from the skin, but also from the mucous membrane of the pharynx, which was also inflamed. In the diffused inflammation of the fauces in cynanche maligna, we have frequently similar formations of matter deep in the neck, from extension of disease from the mucous to the cellular tissue. In the third class of cases, the disease occurred in the habits broken down by want, intemperance, age, or previous organic disease of long standing, and sinking occurred very early, unless prevented by stimulants. In such cases, there was less redness and heat of surface, the affection had more of the character of erythema, with a well defined and raised irregular border; the pulse was quick and weak, the accompanying fever of the typhoid character, the tongue more or less loaded, dry and brown, or red, dry and scabrous near the apex, with elongated papillæ, the rest of its surface much loaded: this latter condition of the tongue generally indicated more or less of gastric irritation.

It is in this form of the disease, that on examination after death, we constantly find evidence of the previous existence of inflammation of the cerebral membranes, of the pulmonary, and of the intestinal mucous surfaces, the cutaneous affection being in fact but a small part of the disease. Severe rigors and diffuse inflammation of the throat, of a dusky red color, with patchy deposition of lymph, and more or less of sloughing, as in cynanche maligna, preceded several severe

11*

cases of erysipelas of the head and face. When the scalp was attacked the inflammation generally became diffused, but occasionally, was limited pretty exactly to one half; when this got well, it was usual for the opposite side to become affected. Delitescence, or disappearing of the inflammation from one part to re-appear in another, was of frequent occurrence. There was a great tendency to the erratic form, particularly in the old, or in persons of broken-down constitution."

An attack of erysipelas was sometimes beneficial in chronic and obstinate cutaneous diseases, as lupus. When the epidemic was very prevalent, there was a remarkable tendency to diffuse inflammation of the digestive tube, without any accompanying erysipelas of the skin. The disase was then rapid — the prostration great. Dr. M'D. lost two patients in this way. The first patient was attacked with diarrhœa on the 4th of March, and died on the 12th, having exhibited tenderness on pressure of the abdomen, bloody stools, with muccus, etc. On examination, there was found diffuse erysipelatous redness of the mucous membrane of the stomach and bowels throughout — the redness being most intense near the termination of the ilium, where shreds of coagulable lymph were thrown out, abrasions and ulcers being also apparent.

Some practitioners, says our author, doubt or deny the existence of morbid appearances after death by erysipelas. He is not one of these. The fatal cases are generally those where the head and face have been effected, and where the last scene is characterized by coma. In these, he usually found much sanguineous congestion in the different tissues, from the skin to the brain — serous infiltration of the loose cellular substance under the scalp, and sometimes sero-purulent fluid — pericranium vascular and thickened — dura mater vascular — arachnoid sac containing a serous fluid — white or milky appearance of this membrane between the convolutions of the brain, with abundant subarachnoid effusion into the cellular tissue of the pia mater — sinuses, cerebral veins, jugulars, and right side of the heart much congested — cerebral substance firmer or softer than natural, showing numerous bloody dots.

In most cases of protracted erysipelas, inflammation of different portions of the gastro-pulmonary mucous membranes was found — especially of the stomach, bowels, and bronchiæ — the inflammation being most intense at the bifurcation of the trachea, with frothy, sanguineous effusion into the smaller ramifications. Gastritis was indicated by epigastric tenderness, vomiting, ardent thirst, loaded tongue, dry, red, and scabrous near the apex — rapid pulse, etc., the strength being very variable. Muco-enteritis was known by abdominal tenderness, of variable extent — tympanitic distention, diarrhœa, the pulse frequent but little disturbed, and the alvine discharges being mucous and gelatinous, with blood occasionally. When the disease spread to the large intestines, there were dysenteric symptoms.

Metastasis of erysipelas is of rare occurrence, but may take place. The author gives a case where erysipelas of the face suddenly disappeared, and was instantly succeeded by acute brochitis, which quickly destroyed life. We have seen some instances of this kind; but more where the external erysipelas suddenly extends to the membranes of brain, without entirely leaving its original seat in the skin of the face or head.

Our author could not see any proofs of contagion in the present epidemic erysipelas; but, from what he had seen in other years, he has no doubt that it is occasionally contagious, both in and out of hospitals. A letter from Dr. Brereton to the author corroborates this opinion, by his experience in the temporary fever and dysentery hospital. We think that few hospital surgeons or physicians can doubt the occasional contagiousness of erysipelas.

"The treatment adopted in the first class of cases, was general or local bleeding, frequently both were employed; incisions were made when there was much tension, heat, and throbbing: calomel, followed by some of the neutral salts, when the stomach was irritable, purgative enemata, and saline effervescing draughts. If the disease did not yield, and symptoms of inflammation of the internal organs manifested themselves, calomel in doses of two or three grains, combined with small doses of opium, was exhibited every fourth or sixth hour, until the mouth was decidedly affected: and cases threatening a fatal termination, when once ptyalism was effected speedily ended satisfactorily. The mercurial affection of the mouth was in general easily controlled by a gargle of the solution of the chloride of lime or soda, with syrup and water; or by the free application of a lotion of nitrate of silver, twenty or thirty grains to the ounce. The vomiting (although in general sympathetic, in some cases obviously depended upon gastritis) was combated by leeching and blistering the epigastrium, and by the exhibition of calomel and opium, with abstinence from all food; drink in sips merely was allowed.

In traumatic erysipelas, emollient poultices and fomentations were employed; some were successfully treated with cloths dipped in cold water, and frequently renewed, or covered over with oiled silk to prevent evaporation: if the inflammation spread, nitrate of silver, in solution, or blisters were employed to arrest or extinguish it. In the second class of cases, general bleeding was inadmissible; leeches, blistering, or the nitrate of silver lotion were generally found sufficient to check the spreading inflammation. Internally, saline purgatives, with small doses of tartarized antimony, or, if the stomach was irritable, blue-pill, followed by saline aperients, and afterwards quinine in small doses, was employed; whenever lesion of the vital organs was threatened, mercury was steadily given to affect the system, at the same time supporting the strength by tonics and stimulants. In the third class of cases, it was necessary to prevent sinking by the early and liberal use of wine or porter, beef-tea, quinine, carbonate of ammonia, and opium; the latter was often necessary to check diarrhœa, but was contra-indicated when there was much cerebral or pulmonary congestion; it was then necessary to excite powerful and extensive counter-irritation, and to stimulate by sinapisms or blisters."

In respect to the local treatment of phlegmonoid erysipelas, every practical surgeon is now aware of the importance of early and free incisions, which speedily arrest the progress of the inflammation, give great relief to the patient, and prevent the sloughing of the fibrous and other tissues, as well as purulent infiltration of the cellular membrane. Puncturing is a bad substitute for incision, and the depth should be in proportion to the extent to which the inflammation has penetrated. In the great majority of cases, it will be unnecessary to go deeper than the cellular membrane. The bleeding is generally profuse, and should be watched, especially in people of intemperate habits or broken-down constitutions. "When (says our author) we observe the rapidity with which the blood flows, it appears pretty evident that there is no stagnation of blood in the inflamed capillaries." To us it appears just the reverse. The greater the engorgement and stagnation, the more rapid the efflux from the vessels when a channel is opened "Qua data porta ruit"—like pent-up winds in the Eolian mountains. Where the disease has been neglected till sloughing occurs, lint, dipped in spirits of turpentine, or in gum elemi and turpentine, should be applied to the incisions, and the whole covered with an emollient poultice.

"Other local applications will also be found of much service in expediting the separation of sloughs and the processes of reparation, as equal parts of castor oil and balsam of copaiba, the Peruvian balsam, or one part of the solution of the chloride of lime or of soda to six or eight parts of water."

When acute erysipelas is limited to the skin, our author recommends leeches. Dupuytren employs blisters. It is often necessary to exhibit tonics to support the strength, and rouse the depressed vital powers. Blisters have been employed with much benefit by our author.

"The local application of nitrate of silver, either in substance or in strong solution, so recommended by Mr. Higginbotham, and by others, has been fully tried by the writer in very many cases. It should be applied not only to the inflamed parts, but also to the neighboring integuments; it should produce vesication, or it is of little service; the smarting from the application lasts but a short time, and is soon followed by decided relief. On the next day, there is generally much diminution of the swelling, the blackened cuticle is much wrinkled, desquamates in a few days; it aggravates the symptoms in phlegmonoid erysipelas, and also occasionally in the superficial form in very irritable habits. Some prefer this application to blisters, which, however, will often be found to succeed when the former has failed. Many object to these plans of treatment, but every practical man must be aware of the urgent necessity of arresting the progress of erysipelas, particularly when it affects the head and face, as an inflammatory state of the brain and its envelopes, accompanied by delirium, and ending in coma, may occur very early if the erysipelas becomes extended.

Lately, I have tried the plan of mercurial inunction, as recommended in the Lancet of July 14th, 1832, page 480, and of September, page 739, and upon the whole am led to consider that it is a most valuable application in this disease. To ascertain as much as was possible, the value of this mode of treatment, it has been employed nearly to the exclusion of other remedies, the bowels being merely regulated, and the diet attended to. In two cases where there was much sinking, tonics and stimulants were combined; in most of the cases, mercury applied in this manner affected the mouth. Understanding that this plan had been used in Mercer's hospital, I applied to Mr. Reid for information, and was favored with the following communication:—

'*York-street, Thursday, June 19th*, 1834.

I lose no time in giving you, from memory, the experience I have of the treatment of erysipelas by mercurial inunction in Mercer's Hospital. I have witnessed the practice both in the idiopathic form, and in the erysipelas consequent to wounds, etc.; it has been used in both species in the head, and also where the extremities have been the seat of the complaint. I think it is more than two years since this practice was introduced amongst us, and was suggested by the cases related in the periodicals, of its success in the hospitals of Paris. It certainly would appear to me from a number of instances, to have considerable power in limiting the extent, and generally checking the progress of the disorder; two, three or four applications have usually sufficed, but the indications of medical treatment have been at the same time followed up. It has been remarked, that no case with us has died, on whom the practice was tried, and that if not in all, in the greatest number of instances, the patients were salivated; from this it would appear, that the absorbents in the diseased surfaces possessed an increased activity. We have now the case of an old man treated by inunction, admitted last Sunday; the erysipelas has disappeared, and the mercurial affection of the mouth is the principal cause of his present illness. This was a very unfavorable case; he received a contused scratch on the forehead a few days prior, to admission; the face, scalp, ears, throat and neck were much tumefied on his admission, and the right arm, from the wrist to above the elbow, similarly affected, though there was no local injury of the limb.'

Many cases are detailed, illustrating the varieties of erysipelas mentioned in this paper; but these we need not analyze. The whole paper is exceedingly creditable to Dr. M'Dowel—and to Dublin surgery in general.—*Medico-Chir. Rev. Jan.* 1835.

10. *Anatomico-Pathological Researches on the Pneumo-Gastric Nerve.* By J. T. H. Albers, of Bonn. *With Observatioos on Diseases of that Nerve.* By Dr. Hankel.—The state of integrity or disease of the pneumo-gastric nerve has, by reason of the important functions it presides over, attracted much of the attention of the more modern pathologists. In all the post-mortem examinations that came before him for nine years, Dr. Albers has never omitted to take notice of the state of this nerve. In the treatise before us, he mentions only those diseases in which the nerve had been most frequently involved.

In the autopsies of 47 persons who had died of hooping cough (some in the second, but most in the first stage of the disease,) he examined both pneumo-gastric nerves in each subject, from their origin to the diaphragm. In 43 there was no change in volume, color, or consistence. Of the 4 others, who were scrofulous and lymphatic, the nerve of the left side was found in one slightly red, and that of the right side of the same color in the three others. This redness was similar to that of the par vagum, in subjects victims to typhus fever, and was always on the side on which the patient commonly lay.

In seven cases of dothinenterite, the right pneumo-gastric was found red in two instances, the left once. This redness affected rather the tissue of the neurilema than the capillaries of the nerve. It disappeared by leaving the nerve some hours in cold water.

In the case of a robust man, æt. 27, who had been attacked in July with dyspnœa, anxiety, delirium, tetanus, and death, in the course of twelve hours, there was nothing presented itself at the dissection but redness and softening of the cervical portion of the pneumo-gastric nerve. It did not lose its color by immersion in cold water, but by exceedingly slow degrees, and then was of a yellowish white. No doubt the color and congestion may be produced on the dead body, by keeping the neck down and the head up, but in such a case the color will be removed by cold water, and the nerve regain its original hue; but this did not occur in the case just quoted.

In fifteen subjects who died of tubercular phthisis, the par vagum was found developed in a remarkable manner; the right was much larger than the left, and in a more palpable degree than is found in the healthy state. This development of the nerve in phthisis is not rare; it has been observed by Swan and Descot.

In two cases of cancer of the æsophagus, the recurrent nerve was entirely destroyed by suppuration; in another case of perforation of the trachea and œsophagus, the vagus was partially destroyed, as was the recurrent.

In a case of medullary cancer of the mediastinum compressing the trachea and œsophagus, the nerve was developed so as to form a little tumor. The cancerous tumor surrounded the nerve. On cutting into its neurilema, there was found a tumor containing a substance similar to the cancerous one.

In a patient who died of intermittent croup, there were found tubercles in the left lung, tuberculous development of the cervical and bronchial ganglions, and intimate union of the pneumo-gastric of the right side with these ganglions. From these facts, Dr. Hankel attributes the attack of croup to the irrigation produced by the softening of these glands, and the death of the patient to the paralysis of the pneumogastric nerve. He is also of opinion that many chronic affections of the chest are owing to organic alterations, tumors, etc. which compress and irritate the par vagum; and that if we find disease in the lung, we too often carelessly omit the minute examination of this nerve.

M. Andral relates a case, in the Nouvelle Bibliotheque, remarkable for its anatomical alterations. The patient was a young man of 24 years old, presenting the following symptoms: swollen countenance; œdema of the eyelids and lower extremities; respiration short, confined, and depending so much on the pectoral muscles, that the lungs seemed paralyzed; inability to remain in the horizontal position; lips and alæ nasi blue; the stethoscope indicated no disease of the heart. Death succeeded suddenly to a fit of dyspnœa. On the examination after death, a portion of the par vagum was found enveloped in tuberculous glands, below which the substance of the nerve was in a state of cartilaginous induration.* — *Ibid.*

11. *Large Doses of the Tartrate of Antimony, after severe wounds and other injuries.* — M. Franc, in his prize memoir, has brought together a multitude of cases of severe injury, to demonstrate the salutary effects of this very powerful remedy. The cases have been drawn chiefly from the cliniques of MM. Delpech and Lallemand, in the Montpellier hospitals, and are valuable alike for the accuracy of their histories and the results of their treatment. The medicine was usually given in full doses — eight or twelve grains being dissolved in water, and a fourth part being given every half hour, till the muscular energy and the arterial and nervous excitement were subdued; and then smaller doses were continued, at great intervals, to keep up the contra-stimulant effects. Several cases of simple and compound dislocations,

*Dublin Journal, Nov. 1834.

in which the reduction was effected with unusual facility, after the exhibition of the tartrate, are mentioned, and, indeed, it was in this class of accidents, and in injuries of the head, that the good effects of the antimonial treatment are chiefly insisted upon. In almost every example, the inflammatory and nervous symptoms were prevented by the timely employment of the tartrate, either with or without the previous abstraction of blood; and even when a train of distressing symptoms had set in before the patient applied for assistance, the relief was uniform and most striking, as soon as the "tolerance" was once fairly established. It is to be observed, however, that, on the whole, this treatment is much more effectual as a preventive than as a curative means.

M. Franc has done a service to his professional brethren, by collecting and arranging the materials of his memoir, for, although the treatment is neither novel nor unknown, we believe that it is not quite so extensively adopted as it ought to be. Many a pound of blood might be saved, if the antimonial anti-phlogistic was duly appreciated on all occasions.—*D'Emploi du Eartre, etc. Montpellier,* 1834.—*Ibid.*

12. *Endermic use of Digitalis in Dropsy.*—The narrator of the following cases assures the profession, that he has been in the habit of employing digitalis, in the endermic method, for the last 20 years, with the most gratifying success.

Case 1.—*Anasarca and Ascites—Frictions on the Abdomen with fresh herb—Cure.*

A countryman became the subject of dropsical effusion into the abdomen and subcutaneous cellular membrane, on the disappearance of a "dartrous" affection of the skin with which he had been troubled for some time. The usual diuretic remedies having been tried without effect, the physician had recommended that the limbs should be scarified, and that paracentesis abdominis should be performed: but fortunately, having heard of the good effects of digitalis, used endermically, he resolved to make a trial of the remedy. The fresh herb was well beaten, and blended with some gastric juice, and this mixture was rubbed powerfully on the abdomen. By the fourth friction, the urine had begun to flow so very abundantly, that the patient, fatigued with rising from bed every minute, "prit le parti s'assooir sur le bord du lit, de mettre les pieds sur des chaises, une chaudron a terre." In the course of one night, the abdomen had greatly diminished in size; the diuresis was kept up, though not to the same extent, for several days, and, within ten days, the patient was completely relieved of every dropsical symptom.

Case 2.—*Anasarca after Scarlatina—Frictions with the Tincture—Cure.*

A child five years of age, while recovering from an attack of scarlatina, became affected with ascites. Frictions were ordered to be made on the abdomen, and inside of the thighs, three times a day, with half an ounce of the tincture of digitalis. In three days the flow of urine had greatly increased, and the dropsical effusion was very visibly diminished; the frictions were then only used twice a day, and this treatment continued for about a week—the cure was complete.

CASE 3.—*Ascites—Frictions with the Tincture—Cure.*

A laboring man, much addicted to the use of spirits, consulted M. Chretien in consequence of dropsy of the belly, under which he had labored for some time. No internal medicines were prescribed, and the only remedy was the rubbing in, three times a day, of half an ounce of the tincture of digitalis on the abdomen and on the inside of the thighs. At the same time, the patient was recommended to abandon the use of ardent spirits. The quantity of the tincture was gradually increased to two ounces per diem. The abdominal effusion very speedily began to diminish, and at the end of a fortnight, was quite absorbed.

Twenty ounces, in all, of the tincture were used.

CASE 4.—*General Dropsy—Frictions with the Tincture in conjunction with internal remedies—Cure.*

Dr. Chretien was called to a man, whom he found in so advanced a state of general dropsy, that he considered that it would not be safe to have recourse at once to the digitalis. He ordered a stomachic strengthening wine, and small doses of subcarbonate of iron, with infusion of rhubarb (a combination which, the Doctor tells us, he very highly recommends in debilitated states of the system.) In the present case, however, no benefit was derived from its use, and frictions with the tincture of digitalis were accordingly commenced, in conjunction with the above internal remedies. In the course of a few days, the alvine and urinary secretions were much increased—the breathing, which had hitherto been greatly distressed, was easier, and the distention of the abdomen was very sensibly diminished; the anasarca of the extremities and of the scrotum was still very considerable.

In order to ascertain whether the cure would proceed without the internal means being adopted, they were discontinued for a few days; but, as the state of the patient became decidedly worse, recourse was had to the infusion of rhubarb, with what the French call "safren aperitif de Mars." It required perseverence in the treatment which we have explained, for nearly four months, to effect the perfect recovery of this patient.

CASE 5.—*Ascites—Internal and external use of Digitalis—Cure.*

A woman, who had labored for some years under cough and dyspnœa, become dropsical; the urinary secretion was remarkably deficient, scarcely more than a wine-glassful being discharged in twenty-four hours.

Pills, consisting of digitalis and of assafœtida, were ordered to be taken three times a day, and the tincture of digitalis was to be well rubbed in upon the abdomen. In the course of a few days after beginning the use of these remedies, the flow of the urine was so abundant, that the patient was obliged to get out of bed almost every hour; the general health became also, at the same time, much better, and, in the course of a month, every unpleasant symptom was entirely removed.

*Observations.* The tincture which was employed in the preceding cases was stronger than that in common use; it was prepared by digesting an ounce of the leaves of the herb in three ounces of alcohol. If the experience of other practitioners should confirm that of Dr. Chretien, the endermic method of exhibiting digitalis will be a most valuable improvement in the mode of treating many cases of dropsy.

The reports of his cases are certainly far too brief, and too carelessly drawn out, to satisfy the mind of the critical reader. No allusion is made to the state of the pulse during the continuance of the remedy; but we are led to infer, from the subsequent remarks of the author, that it was always lowered, when administered internally, and that no sensible effect was produced upon it by the external use of the medicine. It has been maintained by Broussais, and other physicians of the physiological school, that the direct and normal action of digitalis is a sedative to the circulatory system, and that, whenever the pulse becomes quickened, or indeed, is not reduced by its use, we may conclude that some gastric irritation exists in our patient; in order that the drug may be able to exert its natural action, the stomach must be in a tranquil state. The experiments which Dr. W. Hutchinson instituted upon himself are the most satisfactory and conclusive, as to the operation of digitalis on the human body, and they certainly confirm the doctrine of Broussais; the pulse was gradually more and more lowered as the medicine was continued, and its dose increased, until it beat only 28 times in the minute; and a gastro-intestinal irritation, accompanied with extreme exhaustion, having then come on, the contractions of the heart became strong and vigorous, and were upwards of a hundred. It is to be remembered, that this experiment was repeated, and the same results exactly were obtained as on the first occasion.—*Revue Medicale.*—*Ibid.*

13. *Detection of the Matter of Miasmata in the Air.*—In a memoir lately read by M. Boussingault, at the Academy of Sciences, this most curious subject of meteorological enquiry is very ingeniously treated. It is well known that miasmata are almost always generated in places where dead vegetable matter is exposed to the conjoined influence of heat and of moisture, and hence, that they are of frequent and perhaps constant occurrence in warm marshy districts, especially if there be any admixture of fresh and salt waters, or if the ground has recently been turned up. The observations of all enquirers agree in representing the evening, soon after the setting of the sun, as the period at which the miasmatic influence is most actively pernicious, and in showing that the deleterious atmosphere rests chiefly in the valleys and low grounds, while the more elevated positions are often quite exempt from its agency. Many years ago Moscate was led, by attending to these circumstances, to believe that perhaps the matter of miasmata might be discovered by collecting and condensing the dew, and submitting it to chemical analysis. His experiments were performed in the rice-grounds of Tuscany; and the results of these were, that the water so treated did very often exhibit flocculi, which had the properties of an animalised substance. Subsequently M. Delille announced that the water which he collected by condensation from the atmosphere in the marshes of Languedoc readily putrefied, and exhibited then flocculi of a highly azotised matter, which, on the addition of nitrate of silver, afforded a purple precipitate.

In 1819, M. Boussingault observed, that sulphuric acid placed in the neighborhood of pools, in which hemp was steeping, quickly assumed a dark color, whereas, at a greater distance from these foci of putrefaction, the change took place much more slowly. At the period of this observation, a pestilential fever was ravaging the district of Ain, where M. B. was residing; and he was hence led to conjecture that the two co-incident phenomena might be owing to the operation of the same agent, and that this agent, from its blackening sulphuric acid, no doubt, contained some carbonaceous matter.

When M. B. visited South America, he repeated these experiments in many places, which were notorious for their pestiferous miasms; but in these warm regions, the atmosphere so teems with insects, that the change in the color of the acid might be supposed to be owing, in part at least, to some of these having fallen into, and being charred by, it. His apparatus, therefore, required some improvement, to guard against this objection, and he acquaints us, that when he was residing at Carthagena, he adopted the following precautions:—

"Soon after sunset," says he, "I placed two watch-glasses on a table in the middle of a marshy plain: in one of these glasses I put a few drops of warm water, for the purpose of moistening its surface, and imparting to it a temperature somewhat above that of the atmosphere; the cold glass was speedily covered with dew: but almost none was condensed on the other one. A drop of strong sulphuric acid was then added to both glasses, and when their contents were slowly evaporated to dryness by the heat of a lamp, it was invariably found that a trace of carbonaceous matter adhered to that glass in which the dew had been deposited, while none could be seen in the other. This mode of experimenting possessed the advantage of requiring but little time for its performance, and if any insect happened to fall into either glass, it could be at once removed with a needle.

By using two glasses, the objection that any particles of dust floating in the atmosphere might have been entangled in the fluids was guarded against; for if the carbonization depended upon this cause, it is evident that it must have affected both glasses equally."

The above-mentioned experiment was repeated by M. B. for several evenings, and uniformly the same results were obtained.—*Ibid.*

14. *Cases of Fracture of the Carpal Extremity of the Radius, with Practical remarks on that Accident.*

Case 1. *Fracture attended with Displacement radiad—Reduction on the 20th day.*

A lady, 69 years of age, fell with violence on the hand: the true nature of the accident was not recognised; it was believed to be only a sprain. As the swelling subsided the deformity of the wrist became more and more conspicuous, and the movements of the joint still more impeded. Wheh she consulted Dupuytren at the end of the third week, the following was the state of the case;—the hand was strongly drawn outwards, or in the direction of abduction: a depression might be felt at the inferior extremity of the radius: the acts of pronation and supination were scarcely possible, and when attempted, caused extreme pain.

The replacement of the fracture was effected by an assistant grasping the forearm and making counter-extension, while M. D. drew the hand powerfully inwards, or in the line of adduction: by degrees the depression became less and less perceptible, and when the reduction was complete, the joint was well secured by the application of splints, which were kept on for ten days, and then re-applied for other three weeks. The cure was most satisfactory, no deformity remaining.

Case 2. *Fracture in a Child—Accident mistaken—Reduction* 29 *days afterwards.*

This patient received the injury by falling from a tree; the surgeon under whose care he was placed, thought it merely a severe bruise; the deformity became more apparent as the swelling declined. The fore-arm being secured by an assistant, M. Dupuytren was obliged to continue his exertions for a length of time before the depression between the fractured ends of the radius was effaced. At length however this was effected, and splints were applied: the application of these was renewed every ten days for three weeks. The cure was complete.

Case 3. *Circumstances similar to those in the preceding case.*

A boy, 13 years old, in consequence of falling from a tree upon his right hand, fractured the extremity of the radius; but this accident was not detected till the beginning of the fourth week after its occurrence. The reduction was quite as difficult as in the preceding case, and fortunately the event was satisfactory, the boy recovering with the perfect use of the hand.

Case 4. *Fracture—Nature of the accident mistaken—False joint formed.*

A middle-aged man came to the Hotel Dieu to consult M. Dupuytren respecting an injury of his wrist, which he had been told, was dislocated backwards. The accident had happened three weeks before, and had been caused by falling on his hand. Several attempts had been made to reduce this supposed dislocation, but the deformity had always returned as soon as the extension was withdrawn.

Dupuytren at once recognised the nature of the injury. The radius had been broken immediately above its styloid process. The motions of the wrist and fingers were extremely painful, and indeed scarcely possible; but it was observed that the broken ends of the bone were freely moveable upon each other, and the Baron therefore inferred that a pulse, or secondary articulation had begun to be formed, no doubt in consequence of the repeated and violent efforts which had been made to reduce the deformity. He advised the patient to go into the Hospital as an in-patient, but this was declined, so that the event of this case is not known.

*Reflections on the preceding Cases.* It is the opinion of Dupuytren and some other excellent surgeons, that genuine simple dislocation of the wrist-joint very rarely, or perhaps never takes place, and that all the cases recorded as such have been so many examples of erroneous diagnosis. We do not at present include those cases, in which the joint, or its capsule have become diseased, as from white-swelling dropsy, etc. Perhaps under these circumstances, the extremity of the radius may become fairly displaced from the convex arch of the carpal bones; but such an accident must be very rare; all that we insist upon is, that it is seldom or never caused by outward violence. The injuries which have been mistaken for it, are dislocation of the ulna, dislocation of some of the carpal bones, fracture of the radius, or of the ulna, or of both bones, and lastly, separation of their epiphyses: by far the most frequent of these, is fracture of the carpal end of the radius. If these sentiments be correct, how countless must have been the instances of mala praxis. The Italian surgeon Paletta, arrived at the same conclusion, and nearly about the same period (1820) as the celebrated Frenchman; the words of the former are—"Suspicari liceat manum e loco penitus promoveri non posse." And again—"Ut diceres cubitum quasi ad id intentum esse, ne manus e sco nixu excidat."

The case recently published by M. Forget, in the Trans. Med. de Paris, as one of dislocation of the radius, we have no hesitation in pronouncing to have been rather one of dislocation of the ulna, and we have the authority of Sir A. Cooper to show that such a mistake may not be uncommon: he says—"Luxation of the ulna at the first sight exhibits the symptoms of dislocation of the hand backwards." Certainly, in M. Forget's case, the symptoms reported do not warrant the conclusion which the author has come to, respecting the nature of the injury.

When in young children the extremity of the radius is broken, or what is more common, when the epiphysis is detached, the diagnosis is often still more obscure, than when the accident occurs in adults; the crepitation is much less distinct, and sometimes indeed scarcely perceptible, in consequence, no doubt, of the less density of the bone, of the greater quantity of blood effused between its extremities and of the periosteum, and indeed of many of the osseous fibres too, having not been lacerated; hence the lateral displacement of the fractured ends is usually less considerable, and hence too that degree of curvature which the wrist sometimes assumes in this accident, when it occurs in children. This last mentioned phenomenon is particularly worthy of attention, because, from not being well understood, it has given rise to a most serious practical error. It has been supposed that the carpal extremities of the bones of the fore-arm may, in young children become suddenly bent or curved without any fracture, or displacement of the epiphysis, by falling with force from a height on the hands: this accident has been denominated "primitive and accidental curvature of the bones of the fore-arm." The prognosis and treatment are thus described in a treatise, by M. Thierry, in a memoir published in 1808.

"Le mal guerit ordinairement en douze ou quinze jours, s'il n'est pas meconnu; mais, si l'on e'y apporte pas les soins convenables, l'avant bras reste difforme, s'atrophie, et le malade ne peut presque plus s'en servir."

"*Traitement.* Extensions tres fortes, mais graduees, et cooptation avec les paumes des mains pour redresser la courbure saillante."

We need not say that, however correct the prognosis and mode of treatment may be described, the diagnosis is quite at fault.

The following case, extracted from the late Professor Wilson's treatise on the Bones and Joints, is admirably illustrative of the truth of our remarks.

"A child, three years old, fell from a considerable height upon the pavement: the head was seriously injured, and the right fore-arm was bent nearly to a right angle. The child died soon after the accident; and on dissection it was found that the curvature of the fore-arm was the effect of a fracture of the bones; the fracture however did not extend through the entire thickness of the bones, the fibres on the posterior surface being not divided, and the periosteum moreover not being lacerated."

From the investigation of the two most celebrated surgeons of the present day, Sir A. Cooper and Baron Dupuytren, it appears, that there are three sorts of fracture of the carpal extremity; that the bone may be broken either right across, or in an oblique direction into the joint of the wrist, or into several pieces, constituting a comminuted fracture.

Now it is chiefly these two last-mentioned sorts, that have been so generally mistaken for dislocation of the wrist; and the error of diagnosis arises in this way; the wrist-joint, having lost the support and stability afforded by the articulating extremity of the radius, is drawn, by the action of the muscles of the fore-arm, from its normal direction, and becomes inclined either to one side or to the other, or forwards or backwards, dragging the fractured portion of the radius with it, and causing it to project, as if it were dislocated: the circumstances too of the absence of crepitation, and of the facility with which the deformity may be removed for the time by extension, tend to confirm the error of diagnosis. It is to be hoped that this practical error may be henceforth avoided by surgeons, as well for their own credit, as for the successful recovery of their patients; for it is to be remembered, that not a few awkwardly anchylosed joints have been the result of what have been supposed to have been cases of dislocated wrist. Sir A. Cooper judiciously recommends that the splints and bandages should be removed at the end of the third or fourth week, according to circumstances, for the purpose of accustoming the joint to gentle movements. Even under favorable circumstances the joint sometimes does not recover its free motion for many months: this inconvenience is most frequent, as we may suppose, after an oblique or comminuted fracture of the radius.—*Archives Generales, Aout,* 1834.—*Ibid.*

15. *Professor Albers on Inflammation of the Spinal Dura Mater.*—The pathognomonic symptoms of this affection are, according to our author's experience, acute pain, stretching to, and felt chiefly in the lower part of the trunk and inferior extremities; convulsive movements of different parts, accompanied with a trembling or tremulous agitation; difficulty in expelling the urinary and alvine discharges; and lastly, a feeling of tightness or constriction round some part of the body. We shall briefly allude to each of these sets of symptoms in detail.

1. The acute pain in the parts situated below the seat of the inflammation was observed in the examples reported by M. Funk, as well as in those observed by Professor Albers. This symptom has been repeatedly noticed in cases of tetanus. The pain is usually accompanied with a sense of pricking and tearing; and these distressing feelings are in most instances referred to the epigastrium, hips and thighs. In very severe cases so acutely sensitive may be certain parts that even the slightest touch causes excruciating pain and cramps. Now this symptom, or rather chain of symptoms, is not observed, when the arachnoid and pia mater, or when the substance of the medulla itself is affected; under these circumstances, after the pain and convulsive movements have continued for some time, a paralytic weakness usually supervenes. On the contrary, paralysis is certainly not a common result or effect of inflammation of the dura mater, unless the disease has involved the parts situated within its sheath.

2. *Convulsive movements.* When the cervical portion of the spinal dura mater is affected, the muscles of the face and neck are usually drawn into irregular contractions, along with those of the chest and abdomen. The upper extremities are affected more tardily, and sometimes not at all, until a few days before death. It is not, however, unfrequent that severe and irregularly returning pains are felt from the shoulders down the arms; certainly the upper extremities are more generally affected than the lower, when the disease is situated in the cervical region. When the whole extent of the cervical section of the medulla is inflamed, the upper extremities are almost certainly affected; but when the inflammation is limited to its inferior portion, they may escape altogether, or exhibit only a trembling movement or agitation, while the lower limbs are all the time contracted with tetanic spasms. Sometimes the arms are spasmodically affected, while the thighs and legs are paralysed. Under these circumstances, we may expect to find on dissection an effusion of reddish serum within the sheath of the dura mater, at its lower part. As long as the quantity of this serum is small, it may act as an irritant on the cauda equina, and may thus cause convulsions of the inferior extremities; but when it becomes more abundant, it induces, in consequence of the compression, partial or total paralysis.

Professor A. remarks, that the tetanic convulsions which attend inflammation of the dura mater are also always of the "tonic," and very rarely of the "clonic" kind. The cause of the persistance and intensity of the spasm is no doubt attributable to the irritation, which the diseased envelope exercises on the contained medulla, being constant and not remitting. "Now, this is not the case when the medulla itself or its immediate coverings are affected. The state of high excitement lasts but for a short time; hence the violent convulsions are either from the exhaustion of the nervous energy, or from the compression on the nervous mass, especially followed by paralytic weakness. On the other hand, this state of paralysis seldom occurs in inflammation of the dura mater, except for a few hours before death."

3. *Tremulous agitation of different parts of the Body.* This symptom is usually most conspicuous in the early stages of the disease when the patient attempts to walk. He finds that he cannot keep his head steady for a moment, and that his arms, body and, and legs are in a constant tremor. This is one sort of the "paralysis tremens" of Dr. Cooke. It may be observed, that the involuntary action of the muscles we are now alluding to, always ceases when the patient is in the horizontal position. As the disease lasts, this symptom becomes less and less distinctly marked, and either disappears altogether, when a favorable termination may be anticipated, or it is succeded by a tetanic stiffness or contraction of the limbs. As soon as tetanus begins to appear, the tremulous agitation ceases.

4. *Difficulty in expelling the Urinary and Alvine Evacuation.* In general, the paralytic affections of the bladder precedes that of the rectum; and, indeed, the latter viscus is sometimes but little affected till within a short time before death. The true nature of the urinary local distress is not unfrequently mistaken at first, and it is only when the bladder has become enormously distended, that the practitioner is made aware of the existing evil.

5. *The feeling of Constriction round the body.* When the cervical portion of the medulla is inflamed, the sense of constriction is usually experienced round the lower part of the thorax, extending from the dorsal vertebræ to the epigastric region. When the lower part of the dorsal, or when the lumbar division is the seat of the disease, this symptom is remarkable, chiefly in a line running almost parallel to the anterior and superior crista of the os ilii. The sense of constriction is not unfrequently accompanied with positive pain; it is always an unfavorable sign, as it indicates the intensity of the inflammatory action. When once established, it is, even under the most fortunate circumstances, very obstinate of removal.

In concluding these remarks on inflammation of the spinal dura mater, we may state, that the functions and faculties of the brain may remain uninjured, under the most serious disease of the medulla spinalis.—*Graefe und Walther's Journal fur Chirurgie, etc.*—*Ibid.*

16. *On the Treatment of Syphilis without Mercury.*—That men should disagree in matters of opinion, in references drawn from facts of premises, is natural and consistent with what might be expected. But that they should differ on the facts themselves, that one man should term an object white, and another affirm it to be obviously black, is a monstrous and seemingly incredible opposition. Impossible as it might appear, the contradiction is too common to be doubted. Where interest sways, or passion blinds, the philosopher may condescend to explain and to excuse. But where these human frailties do not conspire to distort the observation and to warp the judgment, charity herself finds it difficult to offer a favorable solution of the glaring inconsistency.

This general remark may fairly spring from a survey of the statements which amaze and confound the critic and the profession, with respect to the treatment of syphilitic symptoms. One set of observers assert that syphilis requires mercury for its removal;—the other deny that mercury is necessary, nay, they even affirm that the disease is better cured without it. Both parties found their opinions upon observation—upon *facts.* Yet some of those facts must surely lie, for Nature is true to herself. Who shall determine on which side truth is to be found?

We have had much experience in venereal maladies—have made some experiments and witnessed more. Without attempting to arrogate to ourselves the office of the umpire, we may candidly state in general terms the results of the observations we have made. Our readers will attach what importance they think fit to our conclusions, for in litigated questions of this description something must depend upon the weight of character.

We should premise that this article is based upon two papers; one by Dr. Williams, of St. Thomas's Hospital—the other by Dr. Green of Bristol. The first is contained in the Medical Gazette;* the second will be found in the second volume of the Provincial Medical Transactions.

The great point d'appui of the anti-mercurialists is the army medical reports, those reports display a formidable battery, from which the mercurial practice is assailed. Perhaps the most forcible statements in those documents, are contained in the following extract from the paper of Dr. Green.

---

*Medical Gazette, April 12th, 1834.

"'The reports I have next to call attention to, were circulated from the army Medical Department, in April, 1819, and will be found in Dr. Hennen's work; they state as follows—'That since Dec. 1816, to Dec. 1818, there appear to have been treated, for primary venereal ulcerations on the penis, (including not only the more simple sores, but also regular proportion of those with the most marked characters of syphilitic chancre, as described by Hunter and other writers) 1940 cases.' 'That of these 1940 cases so treated, 96 have had secondary symptoms of sorts;' different of these 96 cases of secondary affections, mercury was had recourse to in 12, for various reasons stated in the report. In the 1940 cases of primary symptoms, mercury was used in 65, for reasons also assigned; if we deduct the 65 and 12 cases in which mercury was used, from 1940 reported, 1863 *cases remain completely cured without mercury.* The report goes on to state, 'that the average period required for the cure of primary symptoms without mercury, when bubo did not exist, has been 21 days; with bubo, 45 days.' That the average period for the cure of secondary symptoms, without mercury, has been from 28 to 45 days. '*That every man treated without mercury, has been fit for immediate military duty on dismissal from the hospital.*' In the same period, 2827 cases of primary symptoms were treated with mercury; secondary symptoms occurred in 51 of them. The report states, that in the majority of these instances, there were good grounds for believing that *the constitutional symptoms* '*were more severe and more intractable than when mercury had not been used for the primary sores.*' Two more thus treated *with mercury* were discharged the service, on account of the injury their constitutions sustained from the treatment. The average period required for the cure of primary symptoms, without bubo, was 33 days; with bubo 50 days; and for the cure of secondary symptoms, 45 days. '*Here then* we have *a record of* 1833 *cases, of every form and stage of syphilis, cured without mercury;* and in not one instance did any unfavorable results follow the treatment; foul ulcerations, caries of the bone, broken down constitutions, and a loathsome death, were not here to be seen. The evidence thus presented is too strong to be disputed, and, perhaps, could have been collected from no other source than military hospitals, and in no other practice could the consequences of the treatment be better observed, as, of course, the men remain under the personal observation of their surgeons after they are cured. I need hardly say, that the enquiries were conducted with that fair, candid, and impartial spirit, which should ever mark the investigations of men searching for truth; if any triumph did follow their exertions, it was that of truth over error."

When we add to this the result of the experiments of Mr. Rose, we have laid before our readers the leading statements contained in the medical reports of the army—statements to which the anti-mercurialists constantly refer with confidence and truimph—the triumph, as they tell us, of truth over error.

"The late Mr. Rose, surgeon to the Coldstream Guards, first stated to the profession in this country,* the important fact, that all cases of the primary and secondary symptoms of syphilis could be cured without mercury. For a year and three quarters he treated, at the regi-

* "Mr. Furguson had previously stated in the 4th vol. of the Med. Chir. Trans. that the venereal disease was successfully treated in Portugal, without mercury."

mental hospital in London, all cases of venereal diseases occurring in the soldiers of the Coldstream Regiment of Guards, without mercury; he observes,* I 'have certainly succeeded in curing all the ulcers of the parts of generation, which I have met with in that period, with the constitutional symptoms to which they give rise, without the exhibition of mercury.' Mr. Rose treated more than 120 cases in this way, and no unfavorable results whatever followed the practice; he adds,† 'caries of the bone, and some of the least equivocal symptoms did not occur. Constitutional symptoms were, in most instances, mild, and, in some, so slight, that they would have escaped notice unless carefully sought for.' In not one instance was there that uniform progress, from bad to worse, which was considered an essentital characteristic of true syphilis. Mr. Rose's paper is well worthy of attentive perusal, as well on account of the facts it contains as of the spirit of cautious enquiry evinced in it. Mr. Guthrie next published a paper on the same subject; he had treated nearly a hundred cases of primary sores without mercury, and considers it an established fact, that‡ 'every kind of ulcer of the genitals, of whatever form or apppearance, is curable without mercury.' He considers that, in some cases, a gentle course of mercury will expedite the cure, but does not consider it a specific for the venereal disease.

Dr. Green of Bristol, who quotes and who admires the foregoing reports — who endeavors

To swell the triumph and partake the gale, has his own experience to confirm their accuracy. His modesty induces him to suppose that his testimony is feeble, yet his candor compels him to present it, in the hope and with the object of inducing surgeons to regard the anti-mercurial treatment without distrust.

For the last seven years, he has treated nearly all the cases of venereal that came before him, without mercury. In a few instances, indeed, he has employed this remedy, where the symptoms got into an indolent or chronic state. He "tried the stimulus of mercury" in such cases, precisely as he would have done whether the symptoms were of venereal origin or not, with the view of inducing healthy action in the local disease; or he has given it to alter the secretions from the stomach and bowels, or to improve the general health where it seemed required.

"I have kept an account of 100 cases of the venereal disease, treated by myself, without a particle of mercury used internally or externally. In the selection of these cases, two objects have been kept in view, namely, that the primary sore should, in some degree, possess the characters of the Hunterian chancre, 'a circular, depressed, and sloughy sore, with an indurated base;' and I have preferred taking notes of those cases where patients resided in Bristol, that I might have an opportunity of watching the consequences of the treatment, to ascertain if any symptoms of a contaminated constitution should result from syphilis, cured without its supposed specific."

Our crippled space will permit us to offer no more than a summary of our author's facts — a still more brief account of his opinions.

---

* Medico-Chirurgical Transactions, vol. viii. p. 355.

† Med. Chir. Trans. vol. viii. p. 420.

‡ Ibid. p. 571.

And first of his facts.

1. All the primary sores exhibited more or less resemblance to the Hunterian chancre. They were treated with sedative and astringent lotions, or with simple ointment as one or the other best agreed. The average period which they took to heal was a fortnight to a month. In some of the cases, the local inflammation accompanying the sore required local or general blood-letting; in others, thickening and induration remained for some time aftei the sores were cicatrized, but spontaneously subsided.

2. Of 100 cases of primary venereal sores thus treated, buboes supervened in sixteen. Of these buboes, six suppurated. The buboes which did not suppurate, were removed, on an average, in about six weeks; in two cases, however, a chronic enlargement of the inguinal glands remained for a longer period, one four, and the other seven months; but both of these patients were of decidedly scrofulous habits. The buboes which did suppurate, were healed within two months from their appearance, except one, which remained open four or five months; this patient was also of a scrofulous constitution; ultimately, the whole sixteen cases of bubo were cured.

3. Constitutional affections, of one kind or another, followed in nine cases; these secondary symptoms were, cutaneous eruptions, sore throat, pains in the limbs and periostitis. The affection of the skin assumed varied forms; it was papular in three cases, pustular in two, vesicular in one, vesicular and scaly in two, and in the last it was a mixed form of eruption, papular in the beginning, followed by a general redness, and ending in a scaly condition of the surface. Two of the papular eruptions were so slight, as scarcely to deserve attention. One was a well-marked specimen of "lichen simplex;" it lasted about three weeks. One case of pustular eruption displayed a striking resemblance to small-pox;—in four months it was removed. The two cases of vesicular and scaly eruptions occurred in delicate and strumous persons; one lasted between three and four months—the other nearly seven. The mixed form of eruption was gone in five weeks.

4. Sore throat occurred in four cases; in three it was conjoined with cutaneous eruptions. We will only observe of the ulceration of the throat that it was extensive, superficial, and covered on its surface with a thin layer of adherent lymph.

5. Periostitis occurred in two cases—broadly marked in one, and faintly in the other. In one, the bones of the head were affected; the complaint was obstinate, but yielded at length to counter-irritation. In the other the tibia was the bone attacked; no treatment was required in this case.

6. There was not one instance of iritis.

Dr. Green deems it necessary to mention the unfavorable symptoms that were noticed. They are neither numerous nor grave.

"In one case of tedious cutaneous eruption, the chain of lymphatic glands of the neck became swollen, and they have remained enlarged since. In another case of obstinate scaly and vesicular eruption, the skin remained discolored, in patches, for nearly twelve months."

Such are the facts of Dr. Green. His opinions may be analytically reduced to this:—that it is absurd and false to consider mercury a specific for syphilis. The belief in its specific agency is, he thinks, only worthy of a place in the manual of some Turkish Doctor, or the hoarded secrets of some copper-colored Indian. He denies that mercury has any power whatever over the syphilitic poison itself—a theory, he observes, unsupported by facts, which has never yet been proved, and never will be. Warming as he proceeds, he throws down the guantlet against *all* specifics; "a belief in which is to say the least of it, a very unscientific faith."* To sum up—he conceives that, cæteris paribus, the venereal disease can be better cured without mercury than with it—that worse consequences follow when the latter is employed than when rejected—and, therefore, that its use should in general be abandoned. Yet he owns that it is sometimes beneficial—but *not* as a specific; his hypothesis and facts continue safe, his creed is not alarmed, at exhibiting the mineral, *not* as a specific.

"The cases in which mercury may be employed with advantage, appear to me to be those in which the symptoms get into an indolent state, and become a chronic disease. Here mercury may be of service, if there be nothing in the constitution of the patient to forbid its use. Its chance of doing good in these cases, results not from any specific or antisyphilitic power belonging to the remedy, but from the well known influence mercury possesses of controlling and altering morbid actions, altogether devoid of any specific character; but, if there be any difference between its influence over specific diseases, and those of a different nature, experience tells us it is this, that while mercury alters and subdues simple morbid actions, it alters and sometimes aggravates those of a specific nature, such as the phenomena resulting from the venereal infection."

Dr. Williams comes next upon the stage. If Dr. Green has prepared the bane, Dr. Williams presents the antidote—if the former denounces mercury as a specific—the latter considers its powers undeniable.

His arguments in favor of those powers are brief—they are general deductions, rather than circumstantial statements.

1. Dr. Williams admits, as it is impossible to deny, that syphilitic, that is venereal sores, and, indeed, venereal secondary symptoms, will in certain instances terminate spontaneously. But he contends, that where mercury is not given, the disease is much prolonged, and secondary symptoms are more frequent.

"It is necessary, also, to ensure a favorable result, that the patient should submit to a painfully long confinement to his bed, and not only that he should abstain from every indulgence, but must submit to an exceedingly low or spoon diet. A spontaneous cure, therefore, is a termination little in unison with our habits of active life, and some remedial mode of treatment is not merely desirable but necessary. In the cure, then, of syphilis, mercury in greater, or less doses, and introduced into the system either locally or by the mouth, or by inunction, is unquestionably, according to our present experience, the great, and indeed only specific remedy by which we are enabled to control and subdue the primary action of this baneful poison."

2. The cure of the secondary symptoms Dr. Williams thinks to be a problem. Yet he makes a faint attempt at its solution.

"The problem of the cure of the secondary symptom is not of such

---

* The Doctor's notions and our's on science may, probably, be widely different. His science may consist in something intellectual, metaphysical, and abstract—our's is the homely induction from fact: his goddess may be throned in the æther or the clouds—our's walks amidst the haunts of men; he may deem it unscientific to believe that sulphur is a specific for the itch—our science is unable to dispute or to contemn the humble truth.

easy solution. It has been affirmed that these symptoms are aggravated wherever mercury has been used for the cure of the primary sores: of this, however, I entertain the strongest doubts; for it is agreed that secondary symptoms much more frequently occur when the primary symptoms have been allowed to terminate spontaneously than when mercury has been used, and yet it seldom happens, when there is so strong a tendency to disease, that such disease, when excited, is mild. It is a fact also, that before mercury was used for the cure of syphilis, it took its name from the more hideous of the forms of its secondary symptoms. In Lisbon also, where mercury is little used for the primary sores, every military surgeon admits that more mutilated faces are to be seen than in any other town of the same size in Europe. But whatever may be the fact, it is certain that the cases of secondary symptoms which present themselves at the London hospitals are of great severity, and show little tendency to a spontaneous cure. On the contrary, the long and extreme sufferings of the patients, and their worn and emaciated frames demand, prompt and immediate relief. In the cure then, of the secondary symptoms mercury and sarsaparilla are the only remedies that at present maintain any character.

The medicinal properties of the latter substance, however, are so little determined—the cases in which it is useful so little agreed on—that Mr. Hunter, and also many most eminent physicians and surgeons, have abandoned it as altogether inefficient; and it is hardly an exaggeration to state, that the majority of the profession, in the present day, rely on mercury as the only remedial agent in all forms of the disease.

If we look, however, to the laws which govern other poisons, we find that their different specific actions are only subdued by many and even opposite remedies; and consequently, we might expect that although mercury might be eminently useful in some stages of syphilis, still, that in combating so multiform a disease, many different modes of treatment would be necessary. In paludal disease, for example, as long as the action of the poison is limited to inducing intermittent fever, unaccompanied by any local disease, quinine is a certain and specific remedy. But no sooner are the secondary affections of that poison established, as inflammation of the liver or spleen, than calomel is the specific medicine; quinine being either unavailing or injurious. An equally remarkable change of treatment is required should the poison of scarlet fever fall upon the larynx or peritoneum, or on the synovial membrane of the joints. It will be plain, therefore, that mercury, the great specific in the primary affections of syphilis, is of doubtful efficacy in its secondary affections; and every practical physician knows how often this powerful remedy totally fails in relieving many of the most distressing forms of the disease."

Such are the arguments adduced by Dr. Williams, in his general support of a mild mercurial practice. The remainder of his paper is occupied with some reflections upon nodes and venereal ulceration of the throat. Our business is at present with the question of exhibiting or withholding mercury for the cure of syphilis. We must, therefore, disregard the observations of the Doctor, which have not particular reference to this subject. Yet we cannot agree with some of his opinions, and his paper may furnish, at a future opportunity, the peg on which to hang some critical remarks.

When we look at the present condition of the argument, we find that it assumes the following aspect. On one side it displayed an alluring and imposing array of facts — cases in the mass of a specious character, which subdue the reasoner by their demonstrative appearance. On the other hand, we discover the majority of the profession, and its experienced portion in particular, clinging, perhaps with prejudice, but certainly with pertinacity, to the doctrine which those facts are calculated to subvert. Cherished errors and prejudices are strong, but truth, after a time, is stronger; and it does appear to offer prima facie evidence in favor of mercury, that the anti-mercurialists so far from gaining ground are daily losing it.

There are reasons for believing *that* a keen examination of the Army Medical Reports would not lead the impartial reasoner to conclusions so unfavorable to the value of mercury, as Dr. Green and others believe; *that* mercury was resorted to in cases which had resisted the non-mercurial treatment; *that* out of a certain number of sores promiscuously taken, many will not be syphilitic at all; *that* if sores be not syphilis, mercury given as a specific would be mischievous, and, consequently the comparison of numbers with numbers treated each way is not a fair one; the point at issue being mercury or no mercury for syphilis only; *that* the experiments were made by men, some, at least, of whom were ill-informed, both of the forms and nature of syphilis, and the proper mode of exhibiting mercury; men with no hospital experience, an imperfect education, and the liabilities to error arising from the enthusiasm excited by the support of a novel doctrine; *that* with all these disadvantages — the application of mercury in cases in which it should not be applied, its exhibition in proper cases in an improper manner, the necessary incompetence of many of the experimenters to conduct such vast and such nice experiments, and all the fallacies which we will not name — with all these disadvantages, the careful consideration of the whole of the reports will still leave the balance in favor of mercury.

Perhaps the investigations of the late Mr. Rose were less likely to terminate in error than those of many of his associates. Those investigations are constantly appealed to as decisive evidence against mercury. Mr. Rose became surgeon to St. George's Hospital, and we can state with confidence, for many gentlemen know the fact, that he *abandoned* in latter life the non-mercurial treatment — that he confessed its inefficacy — and employed the remedy which his statements are frequently brought forward to condemn. We may mention an instance, which came, among others, to our knowledge. A young physician contracted a sore upon the prepuce. He went to Mr. Rose. The latter gentleman merely observed, "I would advise you to take a course of mercury." "Oh! but," said the Doctor, "it can be cured without it." "Never mind that," replied the anti-mercurialist; "if you take my advice you'll take mercury." Mr. Rose was an honorable man, and would never have published what he did not believe to be true. He reluctantly changed his opinions, but he did change them.

Mr. Guthrie is adduced as another authority against the use of mercury. We would ask this question: — *does* Mr. Guthrie now pursue the non-mercurial practice? We should fancy that he does not.

The length to which this article has stretched prevents us from offering many observations arising from our own experience. Yet that experience has not been narrow in the venereal disease. In our next number we shall make some more extended remarks upon the subject.

The facts which we have witnessed, and the experiments which we have made, have been sufficient to convince us of the utility, nay, necessity, of mercury. Like many others, we were charmed with the plausibility of the Army Medical Reports—we imagined that the time was come when syphilis could be treated safely and judiciously without a course of mercury. We were soon undeceived. The great opportunities afforded for observation and for practice in the wards of the Lock Hospital speedily disclosed to us the important truth, that whatever might be the case with respect to the venereal disease elsewhere, no doubt could be entertained of its being best cured by mercury *there.* We do not disbelieve the army surgeons, we do not dispute the authority of Dr. Green, but we believe the evidence of our own senses, and that evidence is in favor of mercury.

Our observations may be reduced to the following general statements: —*that* sores possessing the character of syphilis, when treated without mercury, are remarkably prone to occasion secondary symptoms—*that* secondary symptoms, treated without mercury, are in general extremely obstinate, and in many cases appear to promise no natural termination—*that*, upon the other hand, the same kind of sores and the same description of secondary symptoms, yield with comparative facility to mercury, judiciously exhibited—*that* some sores require but little mercury, and some probably require none—*that* some symptoms usually reputed secondary, such as rupia, sloughy ulceration of the throat and nodes, are occasionally benefitted, and as often aggravated by the employment of mercurial medicines—and finally, *that* mercury, properly administered, is not only valuable, but, practically speaking, indispensable for lues. Such are the conclusions we have drawn from observation and experience, in the Lock Hospital and out of it. That observation has been minute and diligent, if it has not been successful; and, without attempting to play the braggart, we will say that our experience has not been inconsiderable. We went to the investigation with a bias against mercury; the inquiry has given birth to a conviction for it.

The fact is, that there is much, very much, to be done in the examination of the venereal disease. We possess no really good work upon the subject. Hunter's is mischievously wrong—the more modern productions are less boldly erroneous. Many syphilitic symptoms are not at all, or imperfectly delineated—other symptoms are described as syphilitic, which are dubious in their origin or character. Mercury is either not used, or it is abused—it is withheld when it would be safety, given when it is destruction. Scylla and Charybdis are on either side, and to avoid the one, the profession too commonly rush upon the other. In short, venereal symptoms are not sufficiently discriminated, mercury is not appropriately exhibited, and the result, as might reasonably be expected, is confusion.*—*Ibid.*

17. *Memoir of Dupuytren.*—In the number of the Lancet for February the 14th, we gave a biographical sketch of the late Baron Dupuytren. We now propose to take a view of him as a man of science,

---

*In the foregoing article, want of space has compelled us to omit our facts, and to mutilate our opinions. In a succeeding No. we shall endeavor to point out the symptoms that require, and the best mode of giving mercury.

and record, while his memory is still fresh, some notice of all that he has done for the advancement of that art, to the perfecting of which his life and talents were incessantly devoted. It is not easy to convey a personal idea of Dupuytren. He was one of those individuals whose countenance always struck observers as emblematic of a mind whose exact character was not expressible in words. The contemplation o. his features left a 'je-ne-scai quoi' impression on the feelings even of the most acute physiognomists,—half pleasurable and half dissatisfied, —a sensation at once of admiration and dislike, for which it was found impossible to account. Dupuytren was a man of middle stature, brown complexion, and strong make. In his youth he must have been extremely handsome. Those who possessed the personal acquaintance of both must have observed some resemblance between Dupuytren and the professor of anatomy in the University of Dublin. The striking magnificence of forehead, expressive of intelligence of the highest order, and the small dark piercing eye which distinguished the one, had their rival in the other,—that eye, oftenest twinkling with playful malice in the one, and in the other darting those stern annihilating glances which rendered the presence of the great surgeon of the Hotel Dieu so imposing, and frequently so oppressive, to those who fell beneath his scrutiny. 'His eye,' says a French author, 'was enough to terrify a Corsair.'

But it was to the peculiar expression of his mouth that the physiognomy of Dupuytren owed its characteristic cynicism and appearance of universal distrust. Viewing the upper part of his face, and particularly his broad fair forehead covered by a thin white *chevelure*, the figure was that of a man imbued with feelings of benevolence and accustomed to exert the most untiring patience. But soon would the impression be destroyed by a sudden curl of the lip, and an almost imperceptible compression of the mouth, a fastidious though polite shrug of the shoulders, —tokens of the mental storm within, which, with calm exterior, he was disdainful to show, refusing to let his fellows become witnesses of any one feeling that governed him. Without the appearance of avoiding society, though present at all the learned meetings of the French capital, at the Faculty, at the Court, at the reunions of private life, Dupuytren was, intellectually speaking, a perfect anchorite. Admired by all, the friend (perhaps) of a few distinguished men, there was not one who could say 'I know him.' We have already hinted at a cause which, to many, explains the secret reason of the cynicism and distrust of his fellow-men that have thrown a shadow over the fairest days of Dupuytren's life. That cause was sufficient to account for even still more remarkable effects; but others attribute his constant ill-temper and *ennui* to a weakness which is common to a class of great men who are not yet sufficiently great to despise the malice of the envious. Dupuytren never forgot a kindness, and never forgave an injury. His ambition was full equal to his talent, and under the coldest exterior he concealed a heart which was sensible to the slightest impressions. He felt conscious of the superiority which he so fully possessed: and to justify his pretensions, he sacrificed all the pleasures and comforts of private life, and condemned himself, as we have heard him say, to 'lead the life of a dog.' 'Above all things avoid being an insignificant man' (*ce qu'il faut craindre avant tout, ce'st d'etre un homme mediocre,*) was one of his favorite maxims, and to escape the chance of a humiliating mediocrity, he devoted every energy of his mind, succeeding to the utmost verge of his resolve, but not without most bitterly experiencing the stings of envy and calumny, nor without nourishing an implacable hatred against the authors of reports which a man of less susceptibility would have treated with deserved contempt.

The life of Dupuytren afforded various examples of the intensity of this dominant morbid feeling, and of the manner in which he avenged himself. How deep and ramified the root hypocrisy had taken in the vicious court of Charles the Tenth, is too notorious to need description. The royal favor there could only be obtained beneath the guise of professed religion. Every one had his confessor, and the worst sinners passed for the greatest saints. With this crowd Dupuytren was accused of mingling, in person and in object, and malice once went so far as to declare that he had dropped expressly from his pocket a little prayer-book within the precincts of the royal apartment. Innumerable epigrams sprang from the alleged incident, but, equally insignificant with the charge, they were soon buried in oblivion. The memory of the affront, however, never passed from the mind of Dupuytren, and years afterwards, on being accidentally called to attend the daughter of a countess, the supposed authoress of the story, he avenged himself by the infliction of treatment the most cruel and heartless on the mother, at the death-bed of her daughter.

The dress which Dupuytren invariably wore was very peculiar. At the Institute of the Faculty, in town or at the court, in summer and in winter, he was always clothed in a little round-cut green body-coat, to which, when he visited the hospital, was added a small green cloth cap, of a cut altogether original. Those who have at any time followed his clinique at the Hotel Dieu, will remember the slow the almost jesuitic pace, with which he entered the amphitheatre, the brim of his green casquette turned from his forehead, the white apron in front, his right hand thrust into the bosom of his coat, and his left constantly applied to his mouth; for no matter in what society he found himself, whether in public or in private, at the hospital or presiding at a concours at the Faculty, Dupuytren had a habit of constantly knawing the nails of his left thumb and index finger, like one who suffers from some intense bodily or mental pain.

When seated in the professor's chair, he never addressed himself to more than a fraction of the audience; his back was turned upon at least three-fourths of the assembly, and he commenced with a low and indistinct muttering, which afforded little indication of the splendid, and on many occasions truly eloquent discourse that was to follow. The most profound silence always reigned in the crowded class which filled the amphitheatre of the Hotel Dieu, as though all were anxious to catch even the first word that dropped from his mouth; and if, during the lecture, any one permitted himself to betray a symptom of *ennui*, one of his searching glances, with a motion of the lip expressive of the most ineffable contempt, covered the thoughtless culprit with shame and terror.

In the wards of the hospital, the originality of Dupuytren appeared even with more relief. On rare occasions he descended so far as to joke with a patient; but towards the students, and even to his own dressers, he was cold, ironical, capricious, tyrannical, to the last degree. Frequently did it happen, on questioning a patient for a few seconds, that if the answers were not given as clearly and precisely as the inquiries, he would punish the unfortunate *malade* by a shrug of his shoulders, and a departure without a moment's further attention to him. Not easily shall we forget the day when the mother of a child whose leg he was about to amputate, having forced her way into the amphitheatre, suddenly interrupted the operation; the self command of Dupuytren left him, and forgetting what was due to humanity,—to a woman and a mother,—he turned out the agonized parent from the room, with a *coup de pied dans le derriere*.

Dupuytren never tolerated the slightest suggestion or contradiction affecting his measures or opinions, and, as we have remarked, his treatment of the pupils who were placed under him in the hospital was marked with the utmost austerity. The number of his dressers at the Hotel Dieu was twenty-six. At six of the clock every morning he called over the list, and no excuse for absence was admitted. More than once he has publicly degraded an *externe* who had disobeyed his orders, or showed some symptoms of insubordination, by tearing off his white apron and other such insignia; and, on one occasion, it is said that he so far forgot himself as to strike the apothecary of the hospital, giving the offended pharmaceuist, however, the honor and 'satisfaction' of a meeting next day in the Bois de Boulonge; but the duel was, we believe, prevented by the police.

It is a matter of sad experience, that talent and integrity alone are rarely sufficient to raise a man to the high posts of honor in a large capital. The candidate must have protection, and a *savior vivre*, without which he may struggle for years in obscurity. Dupuytren was fortunate in both respects. At a very early period of his life, places were offered to him in the hospitals of several large provincial towns, but he always took care to recommend to the post one of the young rivals whose fame or competition might at a future day become troublesome to him. Thus, of five or six competitors who originally opposed him, he succeeded in placing one at Clarmont, one at Rouen, and one at Strasbourg, and he finally vanquished the remaining three—M. Roux, M. Marjolin, and M. Delpech, in the celebrated concours which took place for the chair of Operative Surgery, on the death of Sabatier, in 1812.

Dupuytren owed his appointment to the head surgeoncy of the Hotel Dieu, where he has ruled, the absolute master, for the last sixteen years, to an accidental circumstance, which deserves to be recorded, both as an example and a warning to hole-and-corner surgeons in all quarters. Previous to the year 1817, Pelletan was surgeon-in-chief of the Hotel Dieu. Dupuytren, who served under him as second, soon became an object of jealousy to the old professor. Distrust succeeded jealousy; then followed mystery, and finally, a secret operation, by which Pelletan was completely ruined. In 1817, there was a patient in the female wards of the Hotel Dieu, who was affected with an enormous carcinomatous tumor of the upper arm. The disease extended to the parietes of the chest and to the neck. The blood vessels were altered, and several other unfavorable complications existed. On a consultation, Pelletan advised an operation, but Dupuytren, in a forcible manner, pronounced various reasons against any attempt to remove the tumor. The patient was undecided. In this state of things, Pelletan was imprudent enough to shut himself up with a few favored pupils, and perform the operation in private, without having informed Dupuytren, or any other person who was absent, of his intention. The patient died almost immediately afterwards, and this event was followed by the retirement of Pelletan.

The reputation of Dupuytren as a first rate surgeon was now fully established. His private practice became considerable, and in 1820 the assassination of the Duke de Berri introduced him to court, thus laying the foundation, if not of his professional reputation, at least of the immense fortune which he has left behind him. If report speaks truly, the surgeon of the Hotel Dieu on this occasion, for the first time, lost the *sang froid* and presence of mind for which he was so remarkable, and committed two essential errors,—one as a practitioner, the other as a courtier. In the first place he sounded the wound of the Duke,—a penetrating wound of the chest! In the next he abstained from answering the King when his Majesty addressed to him some question in Latin. Little faith, perhaps, will be placed in the excellence, or even, the existence, of the Latinity of Louis XVIII, for since the time of the scholarly James, the classics have fallen into disrepute at courts. However, here is the anecdote in detail, as told by a man of letters. Immediately after the accident, Louis, who loved his nephew tenderly, entered the sick chamber, surrounded by a crowd of princes and surgeons, burning with anxiety to know the probable issue of the injury, and at the same time anxious to avoid alarming the patient by any imprudent remark. The King turned to Depuytren, whose appearance even then attracted his notice. Nothing would have been more simple than a whisper in the ear of the surgeon, conveying a request for his opinion. But so close an approximation of king and subject as that species of communication would require, was incompatible with the dignity of a royal personage, and so it was regarded by his majesty, who relieved himself from the dilemma, by reducing his question into Latin, presumed to be the language of physicians, and one with which the patient was known to be but slenderly acquainted. But the words fell dead from the royal lips. No answer was returned to them by Dupuytren, whether from indisposition to reply, ignorance of the language, or confusion at the scene, and M. Dubois, who happened to be present, answered for him.

It was, however, rarely indeed that Dupuytren allowed himself to be surprised. If he was excelled in a few particulars by some surgeons,—if, for example, as we admit, M. Roux was quicker and more dexterous at an operation,—Dessault was more brilliant as a professor—Boyer more prudent and humane, and Marjolin more profound,—there was none who could compare with him for impertubility of mind in the midst of accidents and untoward circumstances, none whose eye was more certain, or whose hand was more firm. Like other surgeons, he has made mistakes. He has opened an aneurysm for an abscess, and has cut for the stone when no calculus existed in the bladder; but such errors only gave to Dupuytren an opportunity of displaying his superiority. They never disconcerted him. Thus, upon one occasion, when extirpating a tumor from the neck, he accidentally opened a large vein, and the patient expired in an instant, from the admixture of air with the blood. Without being affected by an accident which would have disconcerted nineteen out of twenty practiced men, he coolly turned to the class at once, to discuss the cause of death in an extemporaneous lecture, which has seldom been surpassed or equalled for the excellence of its matter and arrangement.

It was, indeed, as a clinical professor that Dupuytren obtained the surpassing reputation which placed him at the head of European surgeons. He succeeded, at the Hotel Dieu, the most eloquent lecturer that France ever produced, and in his new office not only sustained the character of the school at its full height, but raised the clinical instruction to a point which must be regarded as little short of perfection. The 'Lecons Orales,' published under his direction, and from which we have translated so many lectures, convey, perfect as they are, but a feeble idea of the rich and well-selected materials which he has been for years submitting, without intermission, to the attention of the pupils of the hospital. Dupuytren was not what is usually called an orator. He seldom had recourse to literary embellishment, or borrowed from the works of others; but his elocution was simple and elegant, his language clear and perfectly correct. Weariness never stole over his audience during the lecture, from the assemblage of useless details, or superfluous repetitions. On the contrary, his discourse, which flowed from him with the ease and fluency of a perusal, was stored with facts selected from his own practice, and arranged with a clearness that showed how perfectly he understood and had studied every branch of the art.

As an operative surgeon he was successful, without being brilliant; indeed it is notorious that he failed much less frequently than his rival at La Charite, M. Roux, who, in spite of his wonderful dexterity, and excellent method of manœuvring the knife, lost at least three patients for every two of Dupuytren. The cause of this difference is easily explained. Surgery is no longer what it was a century ago,—the art of lawfully cutting and hacking the human body. A more rational direction is given to the studies of those who commence their surgical career. These first apply themselves to medicine as the parent art, and regard surgery in its true acceptation, viz: as a branch of medicine, in which the occasional employment of instruments is demanded. How numerous are the diseases awarded to what is called 'surgical practice,' which not only are 'internal,' but are quite beyond the reach of instruments! Regarding surgery in this its true sense, we hesitate not to place the late Baron Dupuytren at the head of European surgery. He operated with great dexterity and with immovable *sang froid.* But his chief qualities consisted in the perfect correctness of his diagnosis, and the admirable manner in which he managed the therapeutic treatment of his patients. In the great majority of all descriptions of cases, the great difficulty is to establish a correct diagnosis, for on that alone can treatment be correctly founded. The one is subjective to the other, and is a secondary branch of the art. In diagnosis Dupuytren was equalled by no surgeon of his time. A few questions, often put in the most careless tone, a single look, the application of the hand on the abdomen, were sufficient to reveal indications, which, assembled in his mind with almost inconceivable rapidity, afforded conclusions to the surgeon that seldom if ever were erroneous. Not that he was infallible; and it was a reproach that he did not evince that frankness of manner and readiness to acknowledge the commission of an error, which should distinguish all men, and especially surgeons. So far from exhibiting a willingness to admit the commission of a blunder, Dupuytren was not ashamed to resort to unblushing falsehood to conceal it. On one occasion, for instance, at the Hotel Dieu, where the intestine had been opened during the operation for strangulated hernia, Dupuytren, when showing the piece to the class, forcibly thrust his finger through the incision, and dilated with eloquence on the curious way in which gangrenous inflammation sometimes *cuts through the intestines like a*

*knife*, although the interne at his side (at the risk, as he himself said, of being kicked) now and then gave the professor a hint that he was mistaken, and that the opening which he demonstrated was not due to inflammation but to the bistoury. Traits of this kind were not unfrequent. The *amour propre* of Dupuytren even pushed him to the publication of inaccuracies where he was certain of being detected. Thus, in his 'Lecons Orales,' and long before them, in 1824, he boasted that the mortality of the Hotel Dieu was reduced to one patient in twenty, one in nineteen, or one in eighteen, as a mean term, but authentic documents, since published by the authority of the Council-General of hospitals, showed that at that very period the mortality amounted to one in fourteen.

As a writer his reputation is neither great nor extended. He was, in fact, so occupied by practical duties that he had not time to write. It was chiefly as a clinical professor that he shone, and during the twenty years that he gave instruction, the clinical school of the Hotel Dieu has produced more brilliant surgeons, and disseminated more new and wholesome ideas of surgery, than any other establishment of the kind in Europe.

If Dupuytren, however, did not himself write, yet his ideas have been taken up and published by others, and it would not be a matter of difficulty to enumerate a number of excellent works, of memoirs which have covered their authors with renown, that were taken from the fertile source of his clinical instruction. The little which Dupuytren has furnished from his own pen, is to be found in the Memoirs of the Royal Academy, and in the Dictionary of Medicine. Amongst the most remarkable we may enumerate, *in anatomy*, Researches on the Spleen, on the Veins of Bones, on Fibrous and Erectile Tissue:—*in physiology*, on the Nerves of the Tongue, on the Motions of the Brain, on Absorption, and on the Influence of the Eighth pair of Nerves:—*in pathalogical anatomy*, Memoirs on the Neck of the Long Bones, on False Membranes, on Amputation of the lower Jaw-Bone, on Ligature of certain Arteries, on Fracture of the Fibula, on Artificial Anus, on Diabetes Mellitus, on Congenital Luxation, and on Retraction of the Fingers. It is said that he has left an unpublished treatise 'on the Diseases of the Glands.'

Besides these permanent 'titles,' Dupuytren has modified a great number of operations, and is the author of several most useful instruments. For example, his *enterotome* is sufficient alone to have immortalized any reputation; indeed we should be inclined to place his operation for artificial anus after that of lithotrity, and it is infinitely more successful. Thus, up to 1834, forty-one operations, the greater part of which had been rendered necessary by gangrene of strangulated hernia, were performed with the enterotome. Of these only three were unsuccessful; the remaining thirty-eight patients were cured without any accident or risk. Since 1824, at least one hundred operations of the same kind have been performed, and with similar results. Dupuytren also invented a double-bladed bistoury for the bilateral operation, a cataract needle, a compressor in cases of hemorrhage, a porte-ligature; and, as we have mentioned, he has introduced excellent modifications of most of the great operations in surgery.

By his death the science has lost one of its most solid ornaments, and the School of Medicine in Paris its most accomplished professor. The void which he has left is immense. Who can fill it? Who can now succeed to that chair with eclat, which has been filled by Dessault, Pelletan, and Dupuytren? That it will even be filled to the best advantage which circumstances admit, we have reason to disbelieve. Intrigue is at work, and there is cause to engender a fear amongst the profession at Paris, that the clinical instruction in the Hotel Dieu will be intrusted to one of the worst clinical lecturers in the capital. M. Roux removed to the hospital on Monday last, and his place will be filled by Velpeau.—*London Lancet.*—*Boston Med. Magazine, May*, 1835.

